# Comparison of morrow procedure and transapical beating-heart septal myectomy in patients with hypertrophic obstructive cardiomyopathy: a systematic review and meta-analysis

**DOI:** 10.3389/fsurg.2025.1666236

**Published:** 2025-10-16

**Authors:** Maxat Zhakayev, Rustem Tuleutayev, Zhanar Nurbay, Marina Izmailovich

**Affiliations:** 1 Department of Cardiac Surgery, JSC “Research Institute of Cardiology and Internal Diseases”, Almaty, Kazakhstan; 2 Department of Internal Medicine, Karaganda Medical University, Karaganda, Kazakhstan

**Keywords:** hypertrophic obstructive cardiomyopathy, surgical myectomy, transapical beating-heart septal myectomy, systematic review, meta-analysis

## Abstract

**Introduction:**

Transaortic surgical myectomy is the established gold-standard treatment for hypertrophic obstructive cardiomyopathy (HOCM). In contrast, the less invasive transapical beating-heart septal myectomy (TABSM) has recently gained attention as a potential alternative, although comparative evidence regarding their clinical outcomes remains limited.

**Objectives:**

To compare the efficacy and safety of surgical myectomy and TABSM in patients with HOCM.

**Methods:**

A systematic search of PubMed, Web of Science, Cochrane Library, and ScienceDirect (January 2014–May 2025) identified 24 observational studies including 3,732 patients (2,824 surgical myectomy; 908 TABSM). The primary outcome was the change in left ventricular outflow tract pressure gradient (LVOTPG). Secondary outcomes included improvement in NYHA class, prevalence of moderate-to-severe mitral regurgitation (MR ≥ 2), short-term (30-day) and long-term mortality, and the rate of postoperative permanent pacemaker implantation. Random-effects meta-analysis and meta-regression were performed.

**Results:**

Both procedures achieved substantial and comparable reductions in LVOTG, with no significant between-group difference (*p* = 0.75). Functional status improved in both cohorts; younger age and higher study quality were independently associated with greater improvement in NYHA class (*p* < 0.05). Residual MR ≥ grade 2 decreased in both groups. 30-day mortality was low and similar between surgical myectomy and TABSM. Long-term mortality appeared lower after TABSM (≈2%) compared with surgical myectomy (≈6%); however, this finding should be interpreted cautiously due to substantial heterogeneity and shorter follow-up in TABSM studies. Pacemaker implantation occurred less frequently after TABSM than after surgical myectomy (≈2% vs. ≈6%; *p* = 0.03).

**Conclusion:**

Both surgical myectomy and TABSM are effective and safe approaches for septal reduction in HOCM. While surgical myectomy remains the reference standard, TABSM represents a promising minimally invasive option, particularly in anatomically complex or reoperative cases. Prospective studies with standardized endpoints are required to guide individualized procedural selection and confirm long-term outcomes.

**Systematic Review Registration:**

https://www.crd.york.ac.uk/PROSPERO/view/CRD420251075522, Identifier CRD420251075522.

## 1 Introduction

Hypertrophic obstructive cardiomyopathy (HOCM) is a genetic disorder with autosomal dominant inheritance, characterized by myocardial hypertrophy together with structural disorganization of myocardial fibers and fibrotic remodeling (1–4). Left ventricular outflow tract obstruction (LVOTO), often caused by systolic anterior motion (SAM) of the mitral valve in the context of septal hypertrophy and altered papillary muscle anatomy, represents a central pathophysiological hallmark and primary therapeutic target (5–9). Approximately one-third of patients demonstrate resting gradients >30 mmHg, which contribute to symptoms and functional impairment (10–12).

Contemporary myosin inhibitors (mavacamten, aficamten) have shown clinical benefits in selected patients but do not eliminate the need for invasive therapy in those with persistent symptomatic LVOTO (13–18). For such patients, surgical myectomy (SM) remains the gold standard, offering durable hemodynamic relief and favorable long-term outcomes when performed at experienced centers (19–25). Evidence from high-volume programs and contemporary guidelines highlights the importance of institutional expertise and structured referral pathways (26–30). In routine practice, the transaortic approach via median sternotomy is the most widely performed surgical myectomy technique (31–33), and its efficacy in eliminating LVOTO is well established (8, 9, 34–36).

As an alternative, transapical beating-heart septal myectomy (TABSM) has emerged. This technique employs a small apical ventriculotomy to access mid- and apical septal segments without cardiopulmonary bypass. Early series suggest feasibility and symptomatic as well as hemodynamic benefit, particularly in patients with non-basal morphologies (37–39).

While both surgical myectomy and TABSM effectively reduce left ventricular outflow tract gradients (LVOTG) and improve symptoms, comparative data remain limited, and potential differences exist regarding mortality, procedural complexity, conduction system injury, and pacemaker implantation rates (40–42).

This systematic review therefore evaluates and compares the published outcomes of surgical myectomy and transapical beating-heart septal myectomy in HOCM, with a focus on echocardiographic and clinical endpoints.

## 2 Materials and methods

The study protocol was registered with PROSPERO, the International Prospective Register of Systematic Reviews maintained by the National Institute for Health Research (43) (CRD420251075522).

### 2.1 Search strategy

An initial search of the PROSPERO database showed no registered protocols for comparable systematic reviews. We systematically searched PubMed, Web of Science, Cochrane Library, and ScienceDirect from January 2014 to May 2025. A comprehensive search strategy combining MeSH terms and free-text keywords was used to identify studies related to the surgical treatment of hypertrophic obstructive cardiomyopathy. The search included the following terms: (“Myectomy” OR “Surgical Myectomy” OR “transapical beating-heart septal myectomy” OR “TABSM” OR “transapical septal myectomy in the beating heart” OR “classic Morrow procedure” OR “Morrow operation” OR “Morrow myectomy” OR “Morrow technique”) AND (“hypertrophic obstructive cardiomyopathy” OR “obstructive hypertrophic cardiomyopathy” OR “hypertrophic cardiomyopathy” OR “Ventricular Outflow Obstruction”).

### 2.2 Eligibility criteria

Studies that met the following criteria were included in this review: (1) cohort studies or randomized clinical trials; (2) included adults aged ≥18 years are included; (3) studies reporting at least two of the following outcomes: LVOTG, New York Heart Association (NYHA) class, mortality, mitral regurgitation severity (MR ≥ 2), or postoperative complications (e.g., pacemaker implantation); (4) were published in English from January 2014 to May 2025.

The year 2014 was chosen as the cutoff to ensure inclusion of studies reflecting contemporary surgical practice and to allow meaningful comparison with the recently introduced TABSM technique. The primary outcome of interest was the change in LVOTG. The following were defined as secondary outcomes: improvement in NYHA class, prevalence of moderate-to-severe MR ≥ 2, short-term (30-day) and long-term mortality, and rate of postoperative permanent pacemaker implantation. To enhance consistency, all follow-up durations were standardized and reported in months. Mortality outcomes were categorized as 30-day (short-term) and the longest available follow-up (long-term). Where long-term mortality data were not available, this was explicitly stated.

### 2.3 Study selection and data extraction

This systematic review was conducted in accordance with the Preferred Reporting Items for Systematic Reviews and Meta-Analyses (PRISMA) guidelines (44). We extracted the following data from all included articles: first author, year of publication, country, sample size, mean age, gender distribution, mean follow-up duration, NYHA class III–IV, baseline and postoperative LVOTG, mitral regurgitation ≥ 2+, 30-day and long-term mortality, and permanent pacemaker implantation. LVOTG were consistently reported at rest; provoked (Valsalva/exercise) measurements were not available. SAM was mentioned in some reports but was not systematically quantified, and therefore was not extracted for pooled analysis.

For studies that only reported median, range, and/or interquartile range for age, follow-up duration, or LVOTG, we converted these values to mean and standard deviation (SD) according to the methods described by Luo et al. (45) and Wan et al. (46). Baseline (preoperative) and longest available follow-up values were extracted for all outcomes of interest. Thirty-day results were collected when explicitly reported.

Two investigators (ZhM and TR) independently extracted data from the included articles. Any inconsistencies were resolved by discussion between the two authors. If necessary, we contacted the corresponding authors of eligible studies for more information.

### 2.4 Risk of bias (quality) assessment

The studies included in this review were assessed for risk of bias (quality) using the ROBINS-I tool, which provides a more detailed evaluation across seven domains of bias in non-randomized studies.

Two authors (ZhM and TR) independently assessed the risk of bias (study quality) after agreeing on the evaluation method. A third author (NZh) calculated inter-rater reliability between the assessors. Cohort studies scoring six or more points were considered to be of acceptable quality and were included in the systematic review.

### 2.5 Qualitative data synthesis

The data synthesis involved organizing the included studies to ensure a clear and systematic presentation of the findings. Studies were sorted chronologically by publication year and by the last name of the first author. To facilitate a structured comparison, the studies were categorized based on the surgical technique applied: surgical myectomy and TABSM. The results were presented in two structured tables. Outcomes included the proportion of patients in NYHA class III–IV at baseline and follow-up, LVOTG, the prevalence of moderate-to-severe mitral regurgitation (MR ≥ 2), short-term and long-term mortality, as well as the rate of postoperative pacemaker implantation. In addition, data on concomitant procedures [e.g., mitral valve surgery, coronary artery bypass grafting (CABG)] were extracted where available.

### 2.6 Meta-Analysis

Analyses were conducted in RStudio (version 4.5.1) using the “meta” and “metafor” packages. Results from the random-effects model were visualized using forest plots. Heterogeneity among studies was evaluated using Cochran's *Q*-test, the *τ*^2^ statistic (between-study variance), and the I^2^ statistic, which quantifies the proportion of total variability attributable to heterogeneity. Influence analysis was also performed to identify individual studies with disproportionate impact. Between-group differences (surgical myectomy vs. TABSM) were evaluated using subgroup analyses. Publication bias was assessed via Egger's test and visualized with funnel plots. A meta-regression was conducted using multiple study-level covariates, including patient age, study quality (ROBINS-I), follow-up duration, sample size, NYHA class improvement, and mitral regurgitation. Bubble plots were generated to visually illustrate these associations.

## 3 Results

### 3.1 Literature search

**Figure 1:**
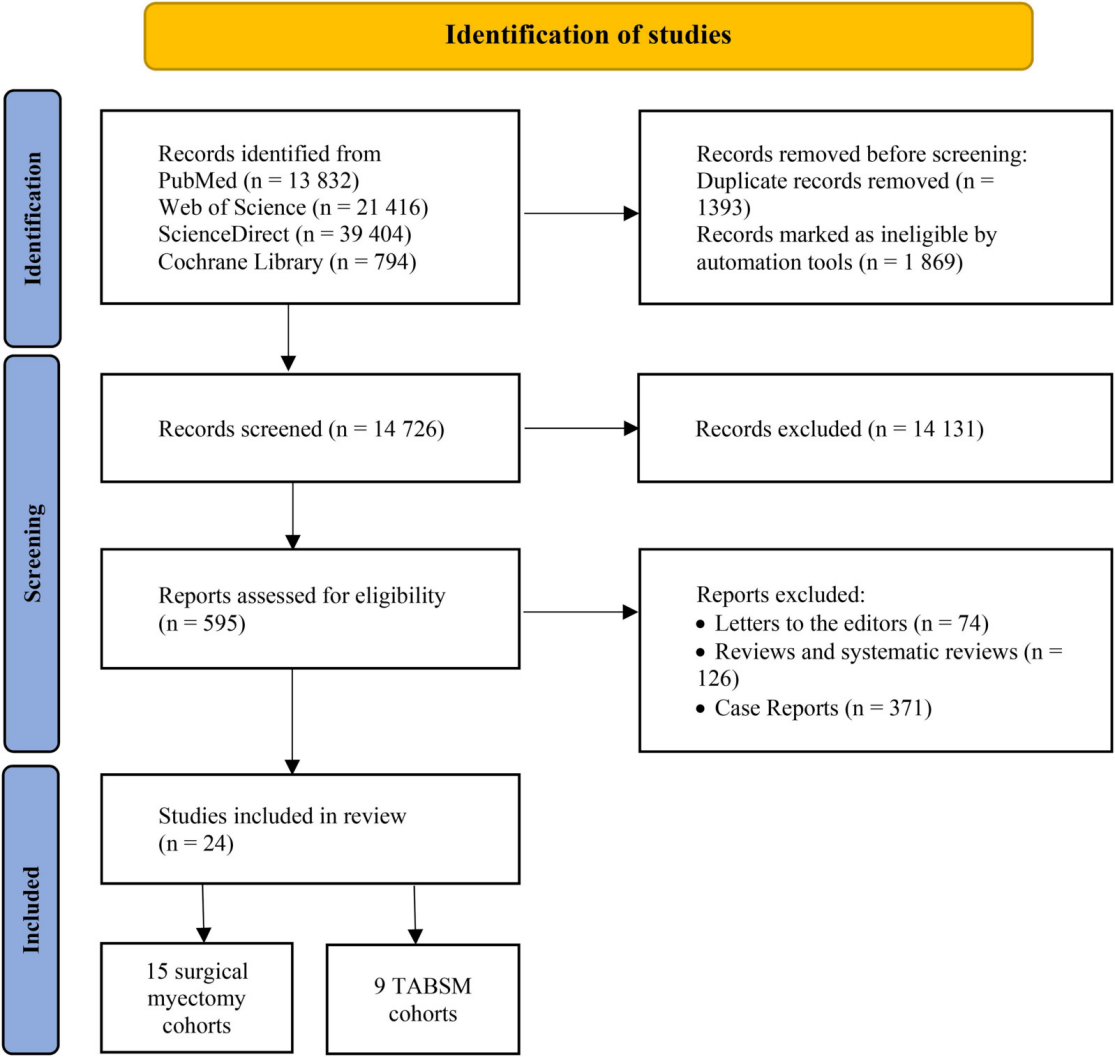
Flow diagram outlining the study selection process according to the PRISMA framework.

We identified 13,832 articles from PubMed, 21,416 articles from Web of Science, 794 articles from Cochrane Library, and 39,404 articles from ScienceDirect databases. After removing the duplicates and records marked as ineligible by automation tools, 14,726 articles remained. Following application of the inclusion and exclusion criteria, 595 studies were retained for full-text review. Of these, 571 were excluded due to duplicate or overlapping data, leaving 24 studies for final analysis. All included studies were observational, with 15 on surgical myectomy and 9 on TABSM (Figure 1).

### 3.2 Study characteristics

Overall, 24 studies were included in this systematic review (Table 1). Fifteen studies focused on surgical myectomy and nine on TABSM. For surgical myectomy cohorts, five studies were from China (908 patients) (47–51), three from European centers (774 patients) (52–54), five from North American centers (1,083 patients) (6, 55–58), and one each from Brazil (33 patients) (59) and Iran (26 patients) (60). For TABSM cohorts, eight studies were from China centers (712 patients) (37, 39, 41, 42, 61–64), one from a North American centers (196 patients) (65).

**Table 1:** Description of the included studies.

Author, year	Country	Follow-up duration, months	Sample size, n	Age, year	Female, %
Surgical myectomy					
Vriesendorp, 2014 (52)	Belgium, Netherlands	94.8 ± 73.2	253	52 ± 16	46
Sedehi, 2015 (55)	USA	164.4	171	48 ± 17.1	51
Parry, 2015 (56)	Canada	52.8 ± 28.8	211	49.0 **±** 16.0	38
Song, 2016 (49)	China	27	16	49.2 ± 15.1	18.8
Vanderlaan, 2017 (57)	Canada	48	150	51.7 ± 14.1	38
Lai, 2017 (50)	China	33.5 ± 24.7	86	50.8 ± 12.0	44.2
Collis, 2018 (54)	UK	60 ± 53.3	272	44.9 ± 16	37.1
Kimmelstiel, 2019 (6)	USA	48.0 ± 34.8	378	52.7 ± 14.7	41.8
Kharbanda, 2021 (53)	Netherlands	50.0 ± 73.8	249	52.7 ± 17.8	42
Meng, 2022 (47)	China	39.2 ± 16.5	502	47.2 ± 11.8	40
Sun, 2022 (51)	China	68.4 ± 35.5	82	48.9 ± 10.4	38.6
Yang, 2023 (48)	China	14.8 ± 4.9	88	44.1 ± 13.1	48
Ayati, 2024 (60)	Iran	81.6 ± 33.8	26	48.7 ± 20.6	53.8
Bruscky, 2025 (59)	Brazil	12	33	49.8 ± 14.0	66.7
Desai, 2025 (58)	USA	12 ± 6	173	51 ± 11	38
Heeringa, 2025 (66)	Netherlands	1	335	50 ± 15	40
Transapical beating-heart septal myectomy					
Sun, 2021 (65)	USA	34.4 ± 38.2	196	48.3 ± 15.6	50.5
Zhao, Li, 2023 (61)	China	3	41	46 ± 2.4	34.1
Zhao, Huang, 2023 (62)	China	3	28	48.4 ± 3.0	28.6
Fang, 2023 (39)	China	3	47	49.1 ± 15.4	36.2
Li 2024 (37)	China	10.3 ± 4.4	120	50.0 ± 14.0	31.7
Li, Wei, 2024 (63)	China	8.7 ± 3.6	61	45.7 ± 18.5	26.2
Lu, 2024 (42)	China	3	105	47.0 ± 18.5	35
Li, Chen, 2024 (64)	China	3	177	50 ± 15.6	29.4
Zhao, 2025 (41)	China	8.9 ± 2.9	133	46.8 ± 16.7	30.8

### 3.3 Subjects

A total of 3,732 patients were included in 24 studies (2,824 patients in surgical myectomy and 908 patients in TABSM). The mean age of the subjects in surgical myectomy group ranged from 44.1 to 52.7 years, and in TABSM group ranged from 46.0 to 50.0 years. The mean follow-up duration of the surgical myectomy group ranged from 12 to 164.4 months, and in TABSM group from from 3.0 to 34.4 months (Table 1).

In the pooled analyses, postoperative values correspond to the longest available follow-up reported in each study, while 30-day outcomes were analyzed separately when reported.

### 3.4 Primary outcome

Preoperative LVOTG ranged from 56 ± 10 mmHg to 108.6 ± 49.8 mmHg in the surgical myectomy group, and from 57.2 ± 28.6 to 92.2 ± 4.0 mmHg in the TABSM group. Both procedures resulted in marked reductions in LVOTG, with follow-up values frequently below 15 mmHg.

### 3.5 Secondary outcomes

The proportion of patients in NYHA class III–IV before surgery ranged from 17.5% to 100% across studies of the surgical myectomy and from 42.1% to 82.0% in studies on TABSM. Postoperative NYHA III–IV class decreased substantially in most studies, often to ∼10%.

Moderate to severe mitral regurgitation (MR ≥ 2) was present from 16.8% to 75.6% of patients preoperatively in the surgical myectomy group, and declined to below 10% in many studies postoperatively. Similarly, MR rates declined after TABSM, though preoperative MR data were reported in fewer studies.

30-day mortality following the surgical myectomy ranged from 0% to 6.3%, with most studies reporting below 1%. Long-term mortality was variably reported across studies. In the surgical myectomy group, available data indicated low but heterogeneous long-term mortality rates, whereas in the TABSM group, long-term follow-up was generally shorter and mortality data were either not reported or limited to single-center experiences. For TABSM, 30-day mortality was ≤2.1%. Although statistical heterogeneity for mortality outcomes was modest, clinical heterogeneity was likely greater due to variation in concomitant surgical procedures across studies.

The rate of postoperative pacemaker implantation after surgical myectomy ranged from 0.4% to 24.2%, with a few studies showing rates above 6%. After TABSM, pacemaker rates were generally lower, between 0.8% and 3.4%, although several studies did not report this outcome.

The clinical outcomes reported across studies are summarized in Table 2.

**Table 2:** Clinical outcomes.

Author, year	NYHA III–IV Pre-op, *n* (%)	NYHA III–IV Follow-up, *n* (%)	LVOTG Pre-op (mmHg)	LVOTG Pre-op, *n*	LVOTG Follow-up (mmHg)	LVOTG Follow-up, *n*	MR ≥ 2 Pre-op, *n* (%)	MR ≥ 2 Follow-up, *n* (%)	SAM Pre-op, *n* (%)	SAM Follow-up, *n* (%)	30-day mortality, *n* (%)	Long-term mortality, *n* (%)	Postoperative pacemaker implantation, *n* (%)	Concomitant procedures, *n* (%)
Surgical myectomy														
Vriesendorp, 2014 (52)	165 (65%)	NA	92 ± 39	NA	8.3 ± 11.9	NA	NA	NA	NA	NA	3 (1.2%)	21 (8.4%)	29 (11.6%)	NA
Sedehi, 2015 (55)	120	26	67.4 ± 43.4	171	11.2 ± 16.4	171	43	17	145 (85%)	104 (61%)	5 (2.9%)	90 (52.5%)	11 (6.4%)	NA
Parry, 2015 (56)	168 (79%)	37 (16%)	64 ± 36	211	5 ± 5	196	58 (28%)	8 (3.5%)	NA	NA	1 (0.5%)	1 (0.5%)	12 (6%)	NS
Song, 2016 (49)	NA	NA	78.45 ± 40.16	16	34.29 ± 21.52	16	6 (37.5%)	NA	11 (69%)	2 (12.5%)	1 (6.3%)	1 (6.3%)	1 (6.3%)	MVR (*n* = 2–4), CABG (*n* = 1–2)
Vanderlaan, 2017 (57)	98 (65%)	NA	67 ± 38	150	11 ± 7	NA	79 (53%)	8 (5.3%)	75 (50%)	0	1 (0.7%)	NA	8 (5.3%)	CABG (*n* = 65), MV surgery (*n* = 17), others (*n* = 59), isolated SM (*n* = 150) analyzed
Lai, 2017 (50)	59 (68.6%) NA	7 (9%)	76.0 ± 43,52,	86	14.97 ± 13.43	77	65 (75.6%)	NA	NA	NA	2 (2.3%)	1 (1.16%)	4 (4.65%)	MVR (37.2% classic vs. 9.3% modified)
Collis, 2018 (54)	NA	NA	71.9 ± 39.6	347	13.4 ± 18.5	NA	NA	NA	NA	NA	3 (1.1%)	15 (5.5%)	27 (9.9%)	Isolated SM (*n* = 272), SM + MV repair (*n* = 33), MVR (*n* = 22), MV repair (*n* = 20)
Kimmelstiel, 2019 (6)	352 (93.2%)	13 (4.2%)	58.0 ± 41.8,	378	NA	NA	127 (34%)	24 (6.3%)	NA	NA	3 (0.8%)	11 (2.9%)	19 (5%)	NA
Kharbanda, 2021 (53)	148 (59.4%)	NA	86.0 ± 22.2	234	13.7 ± 8.1	234	42 (16.8%)	NA			1 (0.4%)	46 (18.5%)	23 (9.2%)	NA
Meng, 2022 (47)	40 (17.5%)	NA	77.1 ± 34.05	502	14.65 ± 13.18	502	NA	NA	NA	NA	3 (0.6%)	9 (1.8%)	2 (0.4%)	SM + CABG (*n* = 44, 8.8%)
Sun, 2022 (51)	0	0	89.1 ± 35.7	82	16.7 ± 12.2	67	NA	NA	NA	NA	5 (6.1%)	3 (3.9%)	2 (2.6%)	NA
Yang, 2023 (48)	62 (70.4%)	9 (10%)	75.3 ± 23.7	88	10 ± 5.2	88	53 (60.23%)	3 (3%)	36 (83%)	1 (2%)	NA	NA	NA	Excluded by design
Ayati, 2024 (60)	8 (30.8%)	NA	67.7 ± 49.6	26	13.3 ± 11.1	26	NA	NA	NA	NA	0	0	11 (42%)	SM (*n* = 26), MVR (*n* = 23), SM + MVR (*n* = 53)
Bruscky, 2025 (59)	33 (100%)	5 (15.1%)	108.6 ± 49.8	33	24.6 ± 46.8	33	21 (63.7%)	NA	28 (85%)	NA	NA	5 (15.2%)	8 (24.2%)	NA
Desai, 2025 (58)	92 (53%)	NA	56 ± 10	136	9 ± 7	136	61 (35%)	9 (7%)	136 (100%)	0	0	NA	NA	NA
Heeringa, 2025 (67)	204 (61%)	23 (7%)	61 ± 30	335	13 ± 12	335	31	6	268 (80%)	28 (8%)	5	NA	NA	NA
Transapical beating-heart septal myectomy														
Sun, 2021 (65)	156 (79.6%)	NA	49.7 ± 40.0	196	9.0 ± 14.1	NA	NA	NA	101 (51.5%)	0	2 (2%)	6	3 (1.5%)	NS
Zhao, Li, 2023 (61)	24 (58.6%)	3 (7.3%)	89 ± 4.9	41	16 ± 1.4	41	32 (78%)	10 (7.3%)	41 (100%)	1 (2,4%)	NA	NA	NA	NA
Zhao, Huang, 2023 (62)	18 (64.3%)	3 (10.7%)	87.2 ± 5.8	28	13.2 ± 1.3	28	28 (100%)	3 (10.7%)	NA	NA	NA	NA	NA	NA
Fang, 2023 (39)	27 (58.7%) NA	0	66.5 ± 37.9	47	20.3 ± 10.4	46	43 (93.5%)	1 (2.2%)	46 (100%)	0	1 (2.1%)	NA	1 (2.1%)	NA
Li 2024 (37)	70 (58.3%)	0	86.3 ± 31.9	120	16.3 ± 9.6	86	79 (65.8%)	6 (5%)	105 (87.5%)	2 (1.7%)	1 (0.83%)	0	1 (0.83%)	No concomitant surgery reported
Li, Wei, 2024 (63)	50 (82%)	0	57.2 ± 28.6	61	11.2 ± 5.6	61	42 (68.9%)	5 (8.2%)	61 (100%)	2 (3.3%)	0	0	2 (3.3%)	NA
Lu, 2024 (42)	56 (53%)	1 (1%)	67.7 ± 44.4	105	13.3 ± 6.7	105	88 (84%)	9 (9%)	91 (87%)	6 (6%)	NA	NA	NA	NA
Li, Chen, 2024 (64)	109 (61.6%)	NA	71 ± 41.5	177	14.3 ± 6.7	177	118 (66.7%)	3 (1.7%)	146 (82.5%)	6 (3.4%)	1 (0.6%)	NA	6 (3.4%)	NA
Zhao, 2025 (41)	56 (42.1%)	1 (0.8%)	92.2 ± 4.0	133	18.6 ± 2.0	133	82 (61.65%)	2 (1.5%)	122 (91.7%)	19 (14.3%)	NA	NA	NA	NA

NYHA, New York Heart Association; LVOTG, left ventricular outflow tract gradient; MR, moderate to severe mitral regurgitation; MVR, mitral valve replacement; MV, mitral valve; CABG, coronary artery bypass grafting; NA, not available; NS, reported, numbers not specified.

### 3.6 Risk of bias (quality) assessment

The results of the ROBINS-I assessment are shown in Table 3. Most studies had a moderate to serious overall risk of bias, primarily due to confounding and missing data. These findings did not materially alter the conclusions of our analysis but highlight the need for caution in interpreting observational evidence.

**Table 3:** Risk of bias assessment using ROBINS-I.

№	Study (First author, year)	Bias due to confounding	Bias in selection of participants	Bias in classification	Bias due to deviations	Bias due to missing data	Bias in measurement	Bias in selection of reported result	Overall ROBINS-I
Surgical myectomy									
1	Vriesendorp, 2014 (52)	Moderate	Low	Low	Low	Moderate	Low	Moderate	Moderate
2	Sedehi, 2015 (55)	Moderate	Low	Low	Low	Low	Low	Low	Moderate
3	Parry, 2015 (56)	Serious	Low	Low	Low	Low	Low	Moderate	Serious
4	Song, 2016 (49)	Serious	Moderate	Low	Low	Moderate	Low	Moderate	Serious
5	Vanderlaan, 2017 (57)	Serious	Moderate	Low	Low	Low	Low	Moderate	Serious
6	Lai, 2017 (50)	Serious	Low	Low	Low	Moderate	Low	Moderate	Serious
7	Collis, 2018 (54)	Serious	Low	Low	Low	Moderate	Low	Moderate	Serious
8	Kimmelstiel, 2019 (6)	Moderate	Low	Low	Low	Moderate	Low	Moderate	Moderate
9	Kharbanda, 2021 (53)	Serious	Low	Low	Low	Moderate	Low	Moderate	Serious
10	Meng, 2022 (47)	Moderate	Low	Low	Low	Moderate	Low	Moderate	Moderate
11	Sun, 2022 (51)	Serious	Low	Low	Low	Moderate	Low	Moderate	Serious
12	Yang, 2023 (48)	Serious	Low	Low	Low	Moderate	Low	Moderate	Serious
13	Ayati, 2024 (60)	Serious	Low	Low	Low	Moderate	Low	Moderate	Serious
14	Bruscky, 2025 (59)	Serious	Low	Low	Low	Moderate	Low	Moderate	Serious
15	Desai, 2025 (58)	Moderate	Low	Low	Low	Moderate	Low	Moderate	Moderate
Transapical beating-heart septal myectomy									
16	Sun, 2021 (65)	Moderate	Low	Low	Low	Moderate	Low	Moderate	Moderate
17	Zhao, Li, 2023 (61)	Moderate	Low	Low	Low	Moderate	Low	Moderate	Moderate
18	Zhao, Huang, 2023 (62)	Moderate	Low	Low	Low	Moderate	Low	Moderate	Moderate
19	Fang, 2023 (39)	Serious	Moderate	Low	Low	Moderate	Low	Moderate	Serious
20	Li 2024 (37)	Moderate	Low	Low	Low	Low	Low	Moderate	Moderate
21	Li, Wei, 2024 (63)	Serious	Low	Low	Low	Moderate	Low	Moderate	Serious
22	Lu, 2024 (42)	Serious	Moderate	Low	Low	Moderate	Low	Moderate	Serious
23	Li, Chen, 2024 (64)	Serious	Low	Low	Low	Moderate	Low	Moderate	Serious
24	Zhao, 2025 (41)	Serious	Low	Low	Low	Moderate	Low	Moderate	Serious

The primary outcome (LVOTG) is presented in the main figures of this manuscript, while analyses of secondary outcomes – including NYHA class, mitral regurgitation, mortality, and pacemaker implantation – are provided in the Supplementary Figures for clarity and adherence to reporting standards.

**Figure 2:**
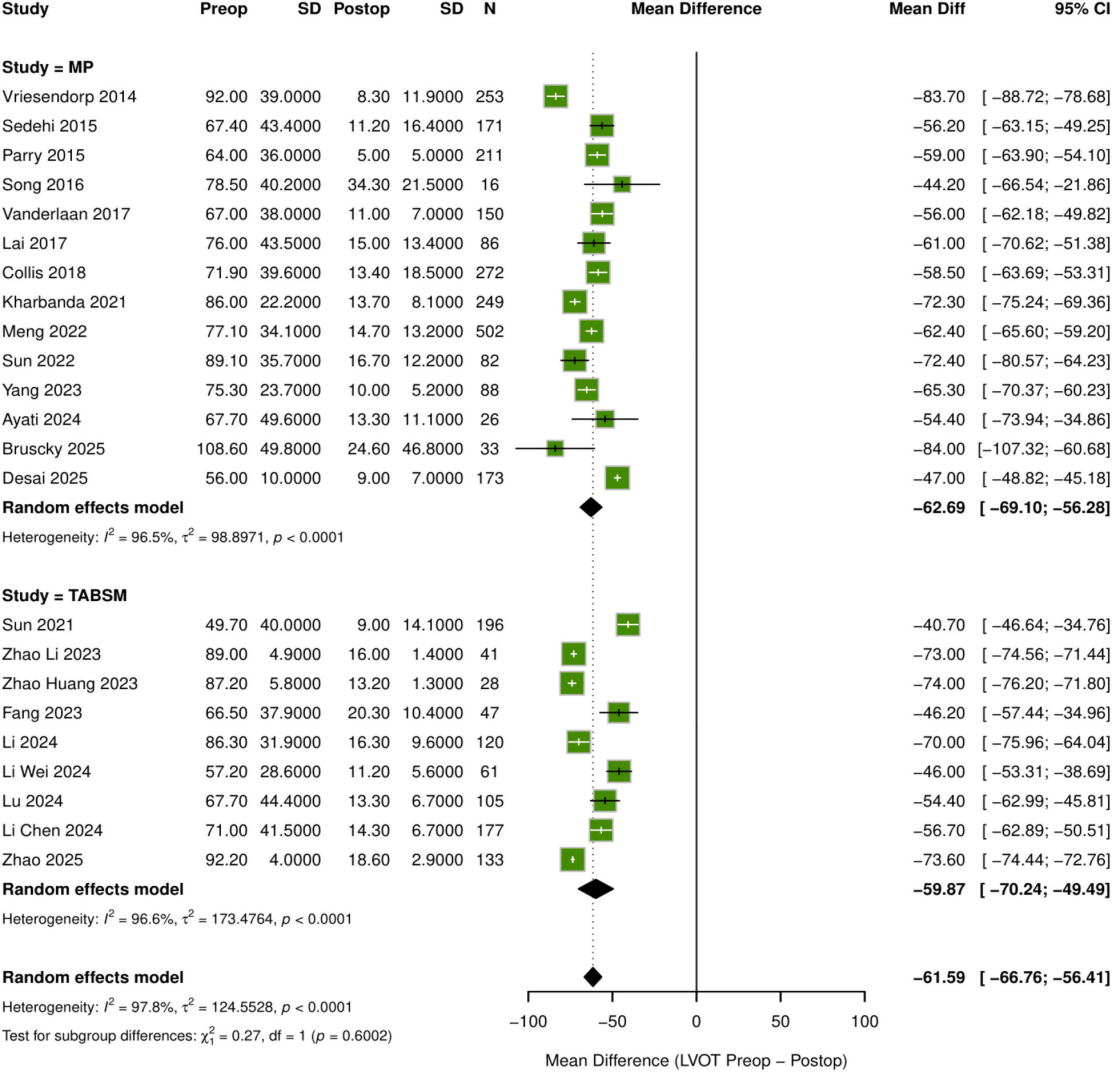
Forest plot: mean differences in LVOT pressure gradient after septal myectomy. MD, mean difference; CI, confidence interval; MP, morrow procedure (surgical myectomy).

Meta-analyses including a total of 24 studies evaluated the efficacy of surgical myectomy on key cardiac outcomes. The pooled analysis demonstrated a statistically significant reduction in LVOTG (mean difference −61.59 mmHg; 95% CI: −66.76 to −56.41; *p* < 0.001), albeit with substantial heterogeneity (I^2^ = 97.8%). Subgroup analyses comparing surgical myectomy and TABSM showed similar reductions in LVOTG with no significant differences between groups (*p* = 0.6002) (Figure 2).

Functional improvement was observed, with a statistically significant reduction in the proportion of patients with NYHA class III–IV symptoms postoperatively (pooled risk difference −0.57; 95% CI: −0.77 to −0.38; *p* < 0.001), although heterogeneity was high (I^2^ = 98.7%). Mitral regurgitation severity decreased significantly after surgery in 14 studies (pooled risk difference −0.54; 95% CI: −0.66 to −0.41; *p* < 0.001). Mortality analyses included 18 studies for 30-day mortality, revealing low event rates with pooled proportions approximately 1%–2% and moderate heterogeneity (I^2^ = 34.5%). Long-term mortality data from 15 studies showed higher mortality rates, with pooled proportions of 6% in the surgical myectomy group and 2% in the TABSM group, and substantial heterogeneity (I^2^ = 84.1%). Pacemaker implantation data included 18 studies, with implantation rates of 6% in the surgical myectomy group and 2% in the TABSM group, showing a statistically significant between-group difference (pooled OR 2.35; 95% CI: 1.07–5.17; *p* = 0.03), with considerable heterogeneity (I^2^ = 82.8%). Across all outcomes, heterogeneity was further assessed using Cochran's *Q*-test and the *τ*^2^ statistic, which confirmed the presence of substantial between-study variance consistent with the high I^2^ values (Supplementary Figure S1).

**Figure 3:**
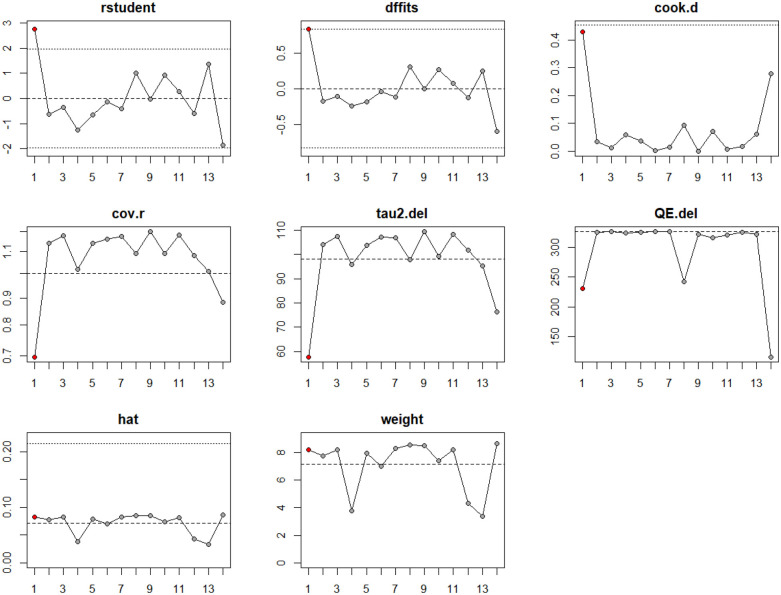
Influence analysis of studies included in the meta-analysis LVOT gradient reduction for surgical myectomy group.

An influence analysis was performed to identify studies with disproportionate impact on the pooled estimate and heterogeneity (Figure 3). Study #14 (Desai, 2025) (58) demonstrated strong influence across several diagnostic plots, including elevated Cook's distance, QE.del, and tau^2^.del values, indicating its major contribution to overall heterogeneity. No other studies demonstrated consistent influence across multiple metrics (Supplementary Figure S2).

**Figure 4:**
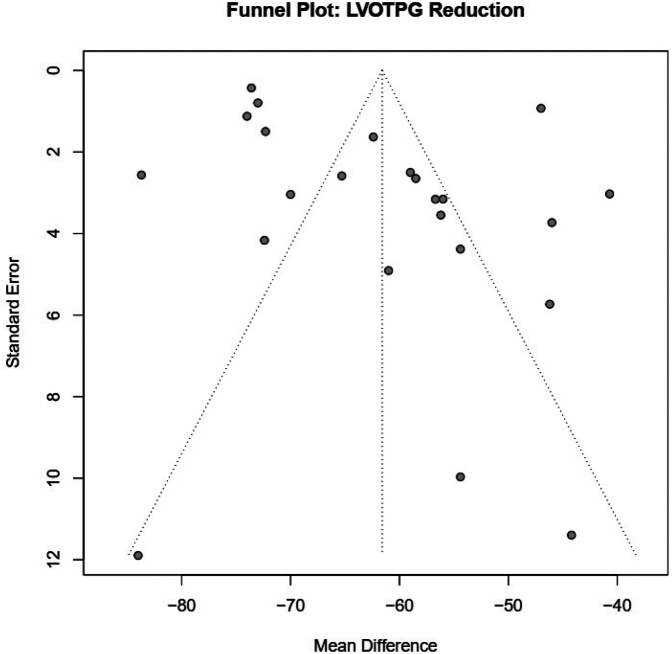
Funnel plots evaluating publication bias for key clinical outcome LVOT pressure gradient reduction.

Upon visual inspection of the funnel plot for studies assessing LVOTG reduction, the distribution appeared broadly symmetrical, although several data points fell outside the expected boundaries (Figure 4). Similarly, for the analysis of NYHA class III–IV improvement and postoperative mitral regurgitation ≥ grade 2, the funnel plots did not reveal marked asymmetry, but publication bias could not be definitively excluded. The plots for 30-day mortality, long-term mortality, and pacemaker implantation also demonstrated a generally balanced distribution of data points, suggesting a low likelihood of small-study effects (Supplementary Figure S3). Overall, while major publication bias is unlikely, the possibility of small-study effects cannot be entirely excluded, and the results should therefore be interpreted with caution.

Meta-regression analyses identified several significant predictors of clinical outcomes. For NYHA class improvement, a multiple meta-regression including age and follow-up duration was significant [QM(df = 3) = 23.83, *p* < 0.001], accounting for 100% of between-study variance. Age (*β* = 0.034, *p* = 0.019) was independently associated with greater improvement, while follow-up duration showed a borderline association (*p* = 0.072). For MR, the multiple meta-regression model was also significant [QM(df = 3) = 21.29, *p* < 0.001], explaining 76.6% of heterogeneity. Follow-up duration emerged as the only significant predictor (*β* = –0.0031, *p* = 0.0024), indicating that longer follow-up was related to greater MR improvement. In the analysis of long-term mortality, only follow-up duration was significantly associated with increased event proportion (*β* = 0.0239, *p* = 0.0073), explaining 33.8% of heterogeneity. Residual mitral regurgitation was also significantly associated with long-term mortality (*β* = 80.21, *p* = 0.025), accounting for 63.9% of heterogeneity. For pacemaker implantation, follow-up duration (*β* = 0.015, *p* = 0.053) showed a borderline association, suggesting a potential influence on the outcome. Other predictors and models did not show statistically significant associations (Table 4).

**Table 4:** Meta-Regression analyses exploring predictors of clinical outcomes.

Outcome	Predictor	*β* (Estimate)	95% CI	*p*-value	R^2^ (%)	k	Model type
NYHA	Age	**+0** **.** **034**	0.006–0.063	0.019	**100**	10	Multiple
	Follow-up duration	–0.0015	–0.0031 to +0.0001	0.072			
LVOT	Age	+4.64	–2.52 to +11.80	0.204	13	23	Single
	Follow-up duration	+0.069	–0.22 to +0.36	0.645	0		
MR	Age	–0.0253	–0.057 to +0.007	0.122	**76.6**	14	Multiple
	Follow-up duration	**–0** **.** **0031**	**–0.0050 to –0.0011**	**0** **.** **0024**			
30-day mortality	Age	–0.0326	–0.2391 to +0.1739	0.757	11.2	18	Multiple
	Follow-up duration	**+0** **.** **0101**	–0.0016 to +0.0218	0.0897			
	LVOT (post-op)	+0.0557	–0.1415 to +0.2530	0.5797	0	5	Single
	NYHA (post-op)	+2.1528	–10.03 to +14.33	0.7291	0		
	MR (post-op)	+15.1708	–6.73 to +37.07	0.1745	**100**		
Long-term mortality	Follow-up duration	**+0** **.** **0239**	**0.0064–0.0413**	**0** **.** **0073**	**33.8**	15	Multiple
	Age	+0.1109	–0.1888 to +0.4106	0.468			
	TAVA Total Score	–0.1496	–0.9197 to +0.6206	0.703			
	MR (post-op)	80.21	10.01–150.41	0.025	63.9	4	Single
	LVOT (post-op)	0.03	–0.83 to +0.89	0.949	0		
	NYHA (post-op)	16.07	–22.7 to +54.8	0.416	0		
Pacemaker implantation	Age	+0.129	–0.117 to +0.374	0.306	5.0	18	Multiple
	Follow-up duration	+0.015	–0.0002 to +0.030	0.053			

Bold values highlight predictors with statistically significant associations (*p* < 0.05).

The bubble plots visually depict the relationships between key clinical predictors and cardiac outcomes across studies. A positive correlation was observed between age and improvement in NYHA class. Follow-up duration is inversely related to mitral regurgitation severity and positively associated with both 30-day and long-term mortality, as well as with pacemaker implantation rates. Residual mitral regurgitation shows a strong positive association with long-term mortality. These visualizations complement the meta-regression analyses and underscore the importance of these predictors (Supplementary Figure S4).

## 4 Discussion

This systematic review and meta-analysis evaluated and compared the clinical efficacy and safety of the transaortic surgical myectomy and the transapical beating-heart septal myectomy in patients with HOCM. A total of 24 studies encompassing 3,732 patients were analyzed, and six key outcomes were examined: reduction in LVOTG, functional status (NYHA class III–IV), moderate-to-severe mitral regurgitation (MR ≥ grade 2), 30-day mortality, long-term mortality, and postoperative pacemaker implantation.

Our findings are in line with large international series demonstrating durable symptomatic relief, sustained hemodynamic improvement, and favorable long-term survival after surgical myectomy. Landmark experiences from high-volume centers such as the Mayo Clinic and Toronto General Hospital have reinforced surgical myectomy's role as the global gold standard for septal reduction therapy (57, 67). In contrast, published TABSM cohorts, primarily from Asian centers and recent Mayo Clinic reports, remain limited in sample size and follow-up, providing emerging but still preliminary evidence regarding feasibility and outcomes (39, 65). By integrating these global data, our analysis places the comparative effectiveness of surgical myectomy and TABSM within the broader international landscape of septal reduction strategies.

The results confirmed that both procedures are effective in reducing the LVOTG, with a pooled mean difference of −61.59 mmHg (95% CI: −67.86 to −55.33; *p* < 0.001; I^2^ = 97.8%), indicating significant and comparable hemodynamic benefit (37, 39–42, 47, 48, 52, 53, 55, 57, 61, 62, 68). Functional improvement was also substantial: the proportion of patients in NYHA class III–IV decreased significantly after surgery (pooled proportion −0.57; 95% CI: −0.67 to −0.47; *p* < 0.001; I^2^ = 98.7%). Notably, meta-regression revealed that younger age (*β* = 0.046; *p* = 0.02) and higher study quality (*β* = −0.12; *p* = 0.03) were associated with greater NYHA improvement, highlighting the importance of both patient selection and methodological rigor.

Postoperative MR (≥ grade 2) was reduced significantly (pooled proportion −0.54; 95% CI: −0.71 to −0.37; *p* < 0.001; I^2^ = 92.7%), with a more pronounced decrease in the TABSM cohort, possibly due to more extensive resection of the mid- to apical septal segments. Although the overall rate of pacemaker implantation remained low, it was significantly higher after surgical myectomy (6% vs. 2%; pooled OR = 2.35; 95% CI: 1.07–5.17; *p* = 0.03), likely reflecting the proximity of the surgical approach to the conduction system (37, 39, 40, 42, 48, 55, 57, 61, 62).

30-day mortality was low in both groups (1.3% for surgical myectomy vs. 0.6% for TABSM; pooled proportion = 0.01; 95% CI: 0.00–0.02; I^2^ = 49.6%), confirming the perioperative safety of both approaches. Long-term mortality was numerically lower in the TABSM group (2% vs. 6%), though heterogeneity was substantial (I^2^ = 77.5%). Meta-regression showed that follow-up duration (*β* = −0.046; *p* = 0.03) and residual MR (*β* = 80.21; *p* = 0.025) were significant predictors of long-term mortality, underlining the clinical importance of complete resolution of mitral regurgitation and ongoing echocardiographic monitoring (37, 39, 40, 42, 47, 52, 53, 55, 57, 62, 68).

Taken together, these findings support the use of both surgical techniques in reducing LVOT obstruction and improving symptoms in HOCM. Surgical myectomy remains the reference standard, particularly for patients with basal septal hypertrophy and well-defined anatomy. However, TABSM may offer key advantages in selected scenarios, including midventricular or apical hypertrophy, reoperations, or when cardiopulmonary bypass is contraindicated. It is important to note that TABSM is a technically distinct procedure requiring advanced intraoperative imaging and highly specialized surgical skills. Some of the included studies represent early institutional experiences with TABSM, and the observed complication rates – including pacemaker implantation – may reflect a learning curve effect. As surgical expertise grows, the complication profile is likely to improve (37, 39, 40).

Additional large institutional and multicenter experiences further support our findings. Kotkar et al. (27) and Hodges et al. (69) reported durable outcomes after surgical myectomy in specialized centers, although these series were not included in our quantitative synthesis due to incomplete reporting of predefined outcomes. More recently, the multicenter registry by Heeringa et al. (66) provided contemporary evidence from 335 patients across 12 hospitals in the Netherlands. Although published after our search cutoff and therefore not included in the meta-analysis, this study confirmed marked LVOT gradient reduction, NYHA improvement, and lower rates of moderate-to-severe MR, with a 30-day mortality of 5% and infrequent complications such as stroke (3%), ventricular septal defect (2%), and reoperation (2%). Together, these reports emphasize that surgical myectomy remains a safe and effective treatment when performed in experienced centers and highlight the importance of institutional expertise and multicenter collaboration.

Looking ahead, the anticipated uptake of cardiac myosin inhibitors such as mavacamten and aficamten may reduce referrals for septal reduction therapy, potentially limiting operative exposure for surgical trainees (70). To preserve expertise in both the surgical myectomy and TABSM, training should increasingly be concentrated in high-volume referral centers, complemented by structured fellowship pathways, simulation-based and virtual-reality curricula, and proctored case series (66, 71). Incorporating competency-based milestones and systematically evaluating the TABSM learning curve will be essential to ensure that future surgeons can acquire and maintain proficiency in these technically demanding operations.

In addition to comparing the surgical myectomy and TABSM, it is important to provide a concise procedural overview of the TABSM itself. This approach is characterized by a transapical incision that enables direct visualization and resection of the mid- to apical septal myocardium under continuous hemodynamic monitoring, without the need for cardioplegic arrest. The use of intraoperative echocardiography and advanced imaging modalities facilitates precise resection, contributing to improved relief of LVOT obstruction and reduction of mitral regurgitation (39, 72, 73).

Furthermore, the surgical management of patients with HOCM extends beyond these two techniques. Alcohol septal ablation (ASA) remains the most widely utilized interventional alternative, particularly in patients who are at high surgical risk or have suitable septal perforator anatomy. Other less frequently performed strategies, including dual-chamber pacing and hybrid surgical–interventional procedures, have been described, although their role has diminished with the refinement of both surgical and catheter-based septal reduction therapies. Contextualizing TABSM within this broader spectrum of septal reduction options underscores its emerging role as a technically demanding but promising alternative, particularly in anatomically complex or surgically challenging HOCM subsets (20, 74–76).

Our findings support a tailored surgical approach for septal reduction in HOCM. While the surgical myectomy remains the benchmark, TABSM offers a minimally invasive alternative with similar efficacy and safety when performed in experienced centers. Preoperative imaging – particularly with three-dimensional echocardiography and cardiac MRI – should be systematically employed to guide anatomical evaluation and procedural planning.

This study has several limitations. First, all included studies were observational, introducing a risk of selection bias and limiting causal inference. Second, inconsistent reporting of concomitant surgical procedures represented an additional limitation. Although these data were collected and summarized in Table 2, incomplete reporting precluded pooled analysis and may have introduced bias when attributing outcomes solely to surgical myectomy or TABSM. Such variability in concomitant interventions may also contribute to clinical heterogeneity in mortality outcomes, beyond what was captured by statistical measures of heterogeneity. In addition, LVOTG were reported only at rest, without provoked (Valsalva/exercise) assessments, which precluded evaluation of latent obstruction and its potential impact on reoperation risk. Similarly, SAM was inconsistently reported across studies and generally limited to descriptive observations, preventing its inclusion in the pooled analysis. Third, considerable heterogeneity was observed in key outcomes (I^2^ > 95% for LVOTG and NYHA class), reflecting variability in surgical technique, follow-up duration, and outcome reporting. Fourth, the lack of individual patient data precluded stratified analyses by relevant clinical variables such as septal thickness, mitral anatomy, or prior interventions. Fifth, definitions of postoperative MR and pacemaker implantation varied across studies and were inconsistently reported. Although meta-regression identified follow-up duration and residual MR as predictors of long-term mortality, other potential confounders could not be analyzed. Sixth, the number of TABSM studies was relatively small compared to surgical myectomy cohorts, which may limit the generalizability of findings. Finally, despite broadly symmetrical funnel plots, several data points fell outside the expected boundaries, and together with the Egger test this indicates that small-study effects and potential publication bias cannot be excluded.

## 5 Conclusion

Both the transaortic surgical myectomy and transapical beating-heart septal myectomy are effective and safe techniques for septal reduction in patients with HOCM. While the surgical myectomy remains the gold standard, TABSM provides a safe alternative. Procedural selection should be individualized based on patient anatomy, institutional experience, and surgical expertise. Future research should prioritize prospective, multicenter randomized trials with standardized definitions and patient-centered outcomes to strengthen the evidence base for surgical decision-making.

## Data Availability

The datasets generated and analyzed during the current study are available from the corresponding author on reasonable request. No datasets were deposited in public repositories.
