# Supplementary material for: Comparison of morrow procedure and transapical beating-heart septal myectomy in patients with hypertrophic obstructive cardiomyopathy: a systematic review and meta-analysis
Source: Front Surg. 2025 Oct 16;12:1666236. doi: 10.3389/fsurg.2025.1666236 (PMC12573290; doi:10.3389/fsurg.2025.1666236)

SAPPLEMENTARY

S1. Forest plot: (A) Risk difference in NYHA class III–IV patients pre- and post-operation; (B) Risk difference in mitral regurgitation pre- and post-operation; (C) Proportion of patients with 30-day mortality; (D) Proportion of patients with long-term mortality; (E) Proportion of patients with pacemaker implantation. MP group – classic surgical myectomy performed through the aortic valve; TABSM group - transapical beating-heart septal myectomy performed via the left ventricular apex. Abbreviations: MD—mean difference; CI – confidence interval; MP - Morrow procedure (surgical myectomy).


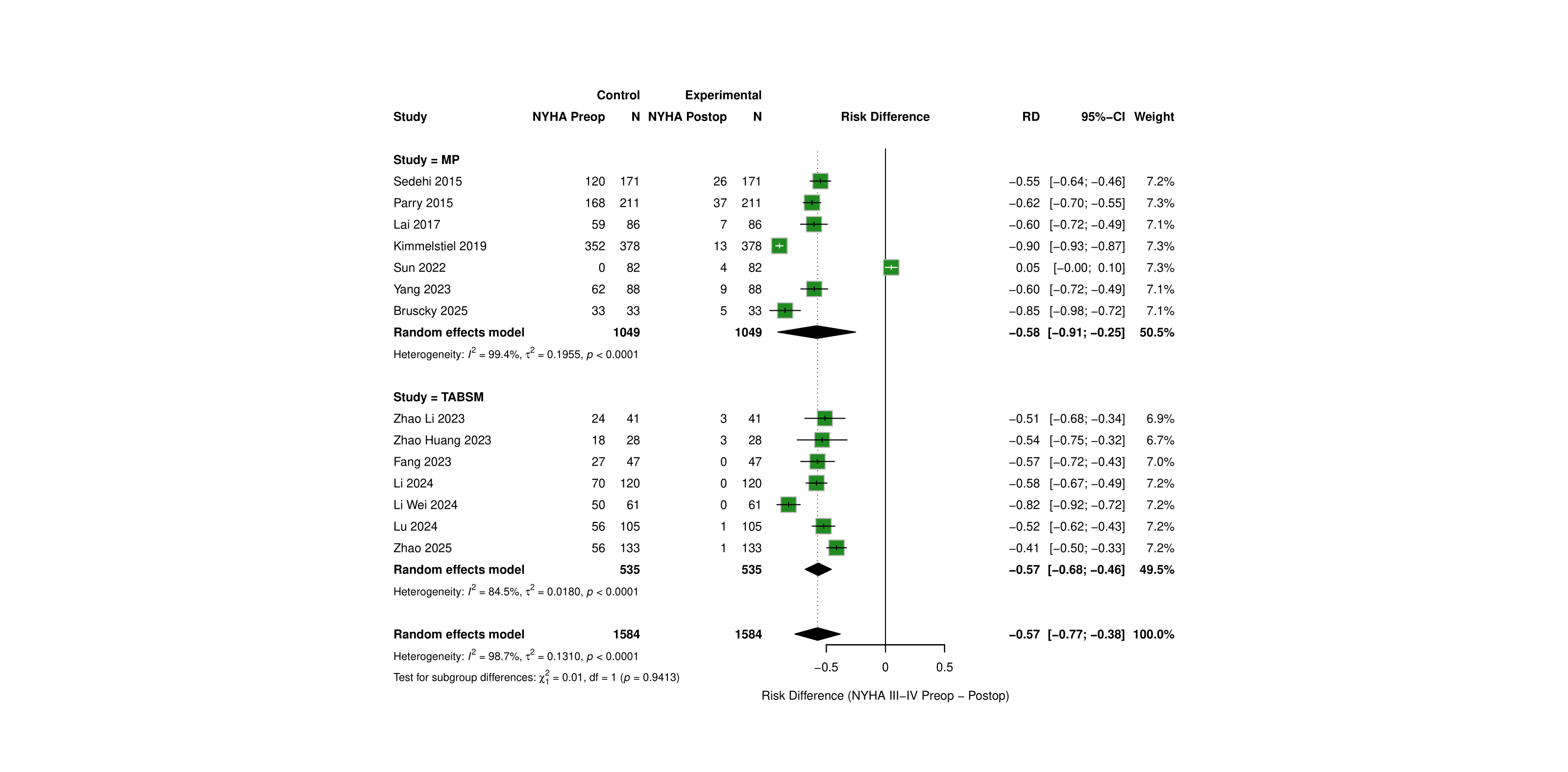


A


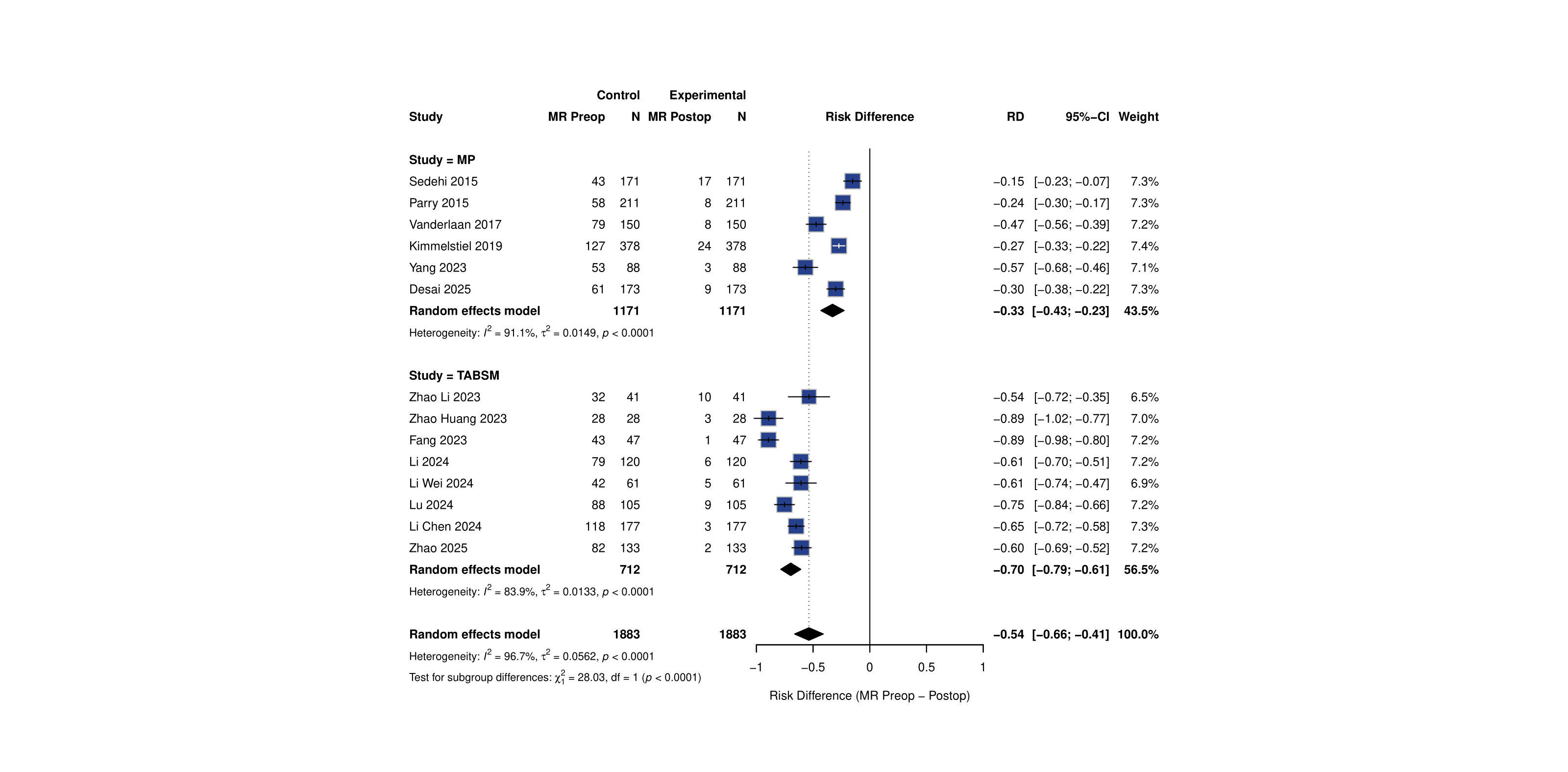


B


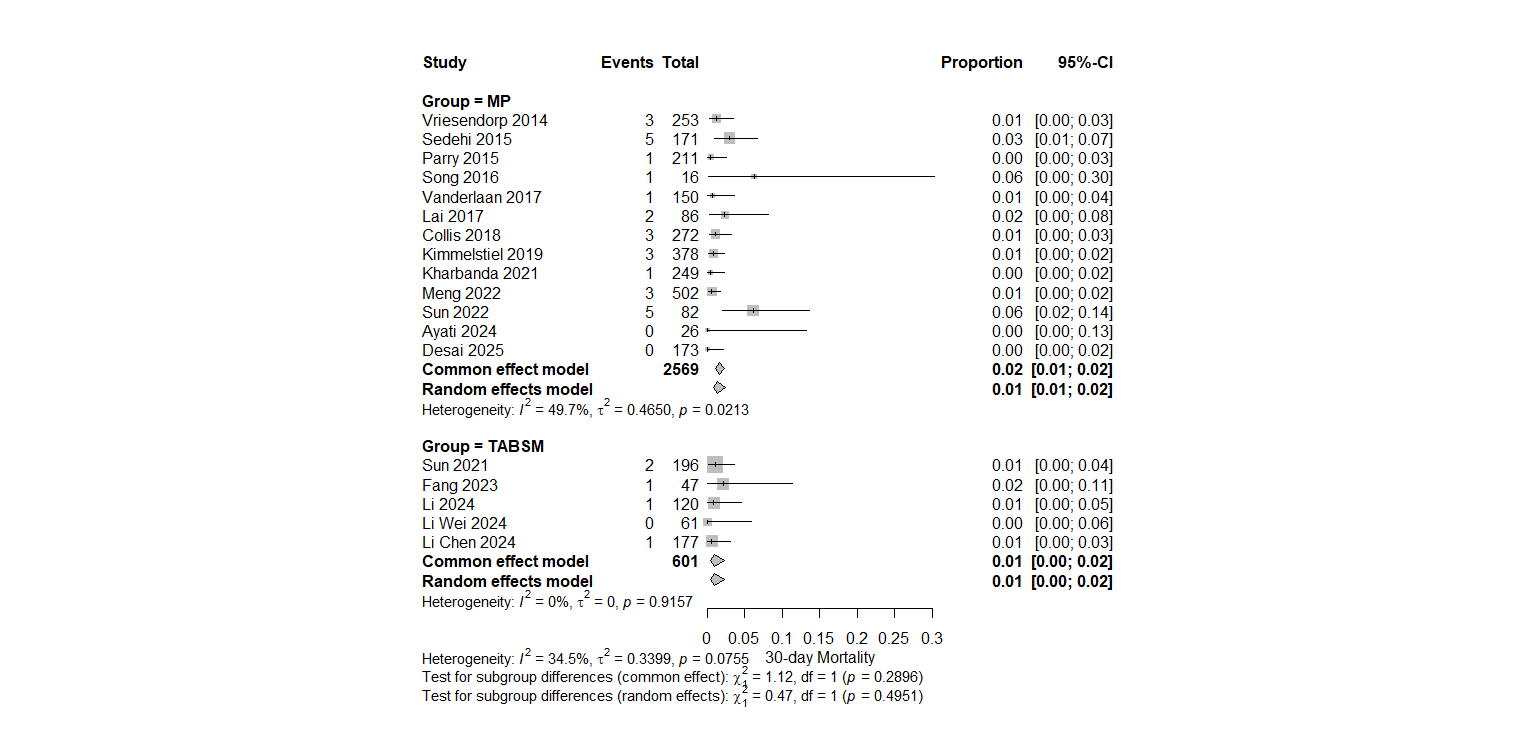


C


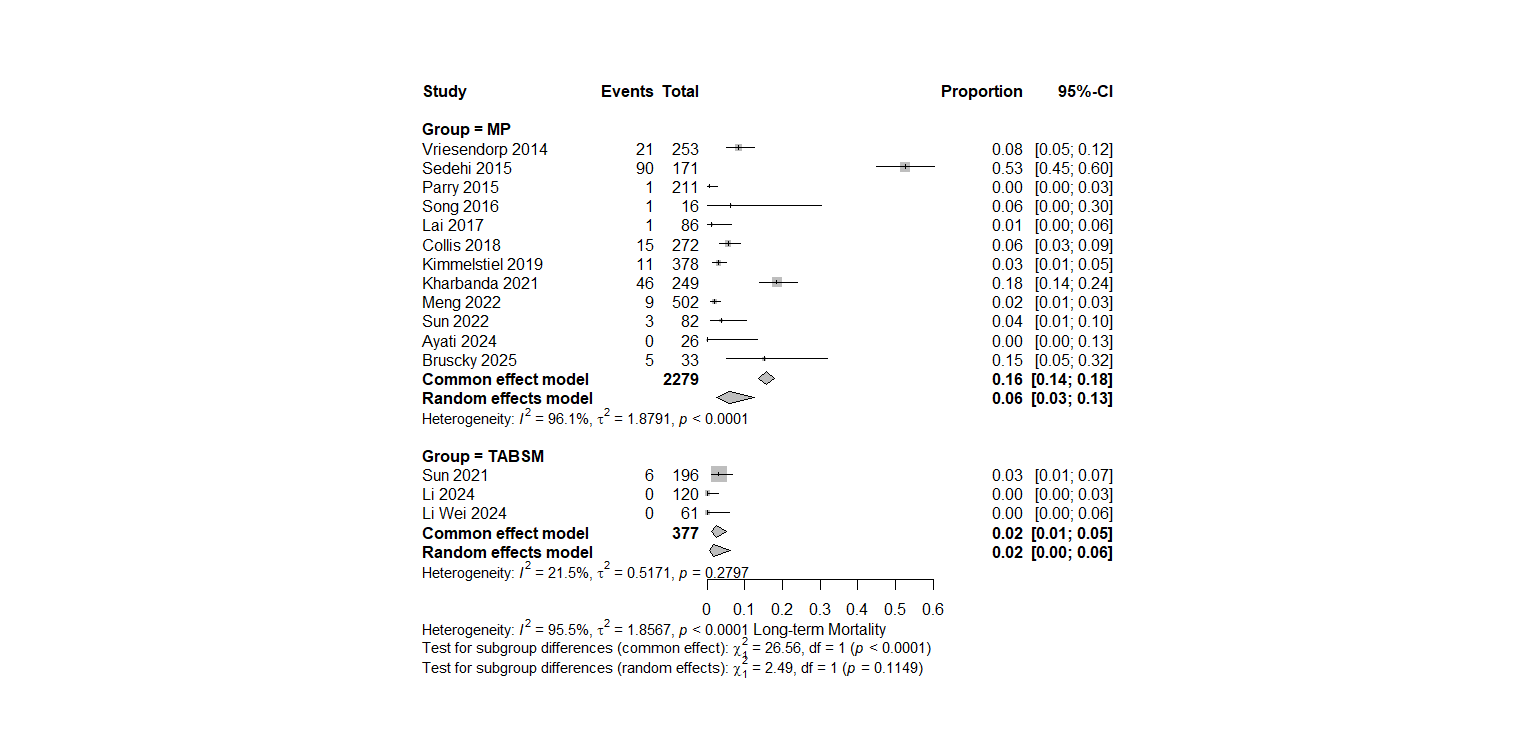


D


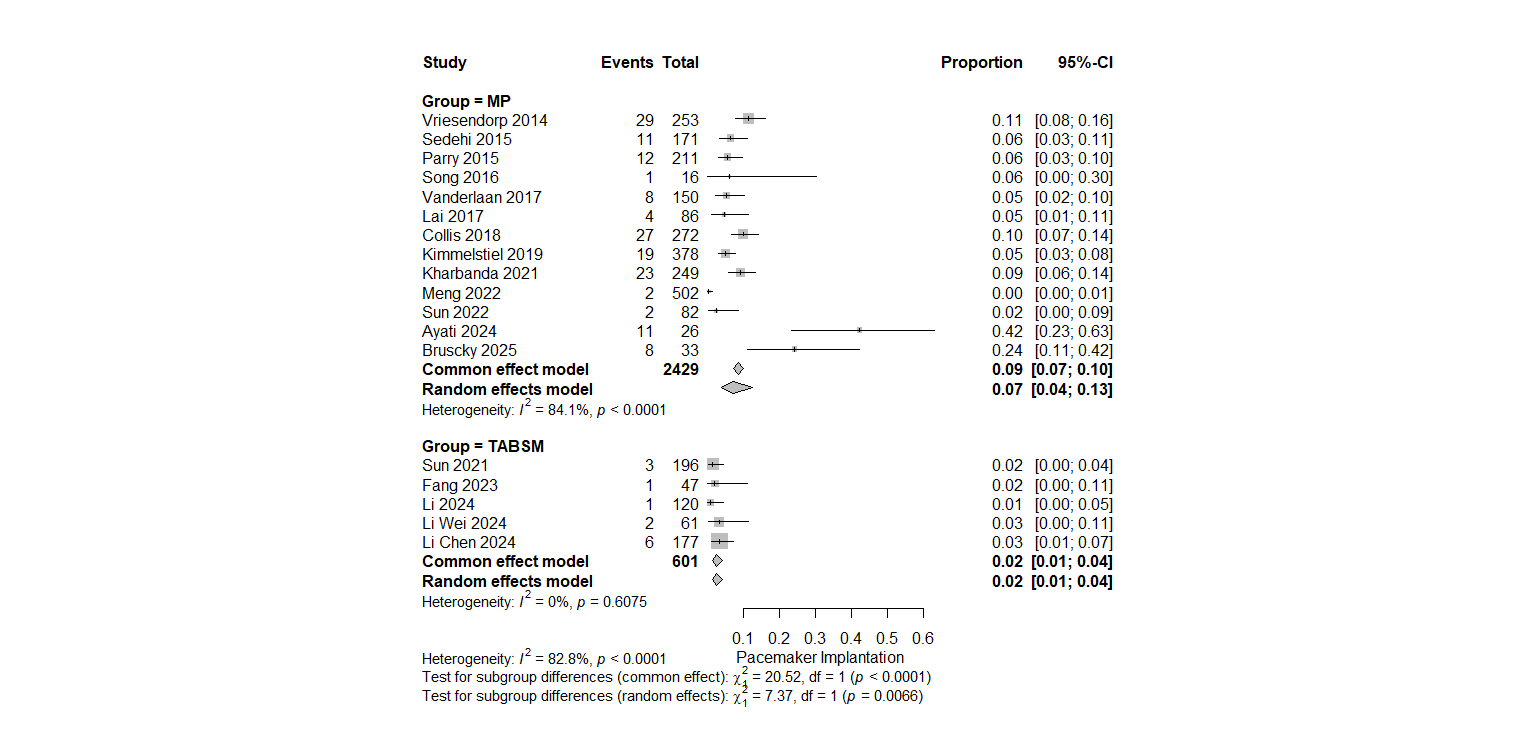


E

S2. Influence analysis of studies included in the meta-analysis: (A) – LVOT gradient reduction for TABSM group; (B)– Reduction of NYNA class III–IV patients in the SM group; (C) – Reduction of NYNA class III–IV patients in the TABSM group; (D) – Reduction of MR ≥ 2 patients in the SM group; (E) – Reduction of MR ≥ 2 patients in the TABSM group.

A
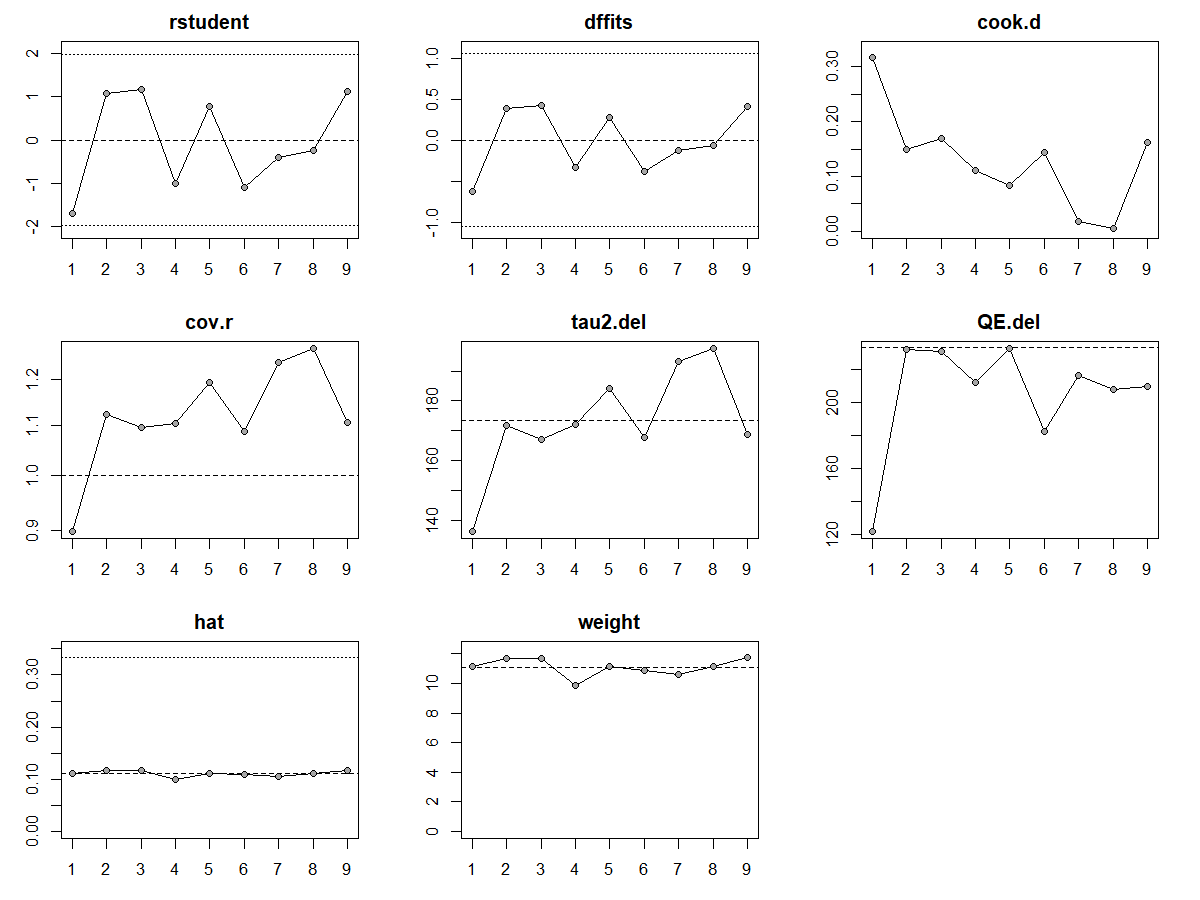
 B
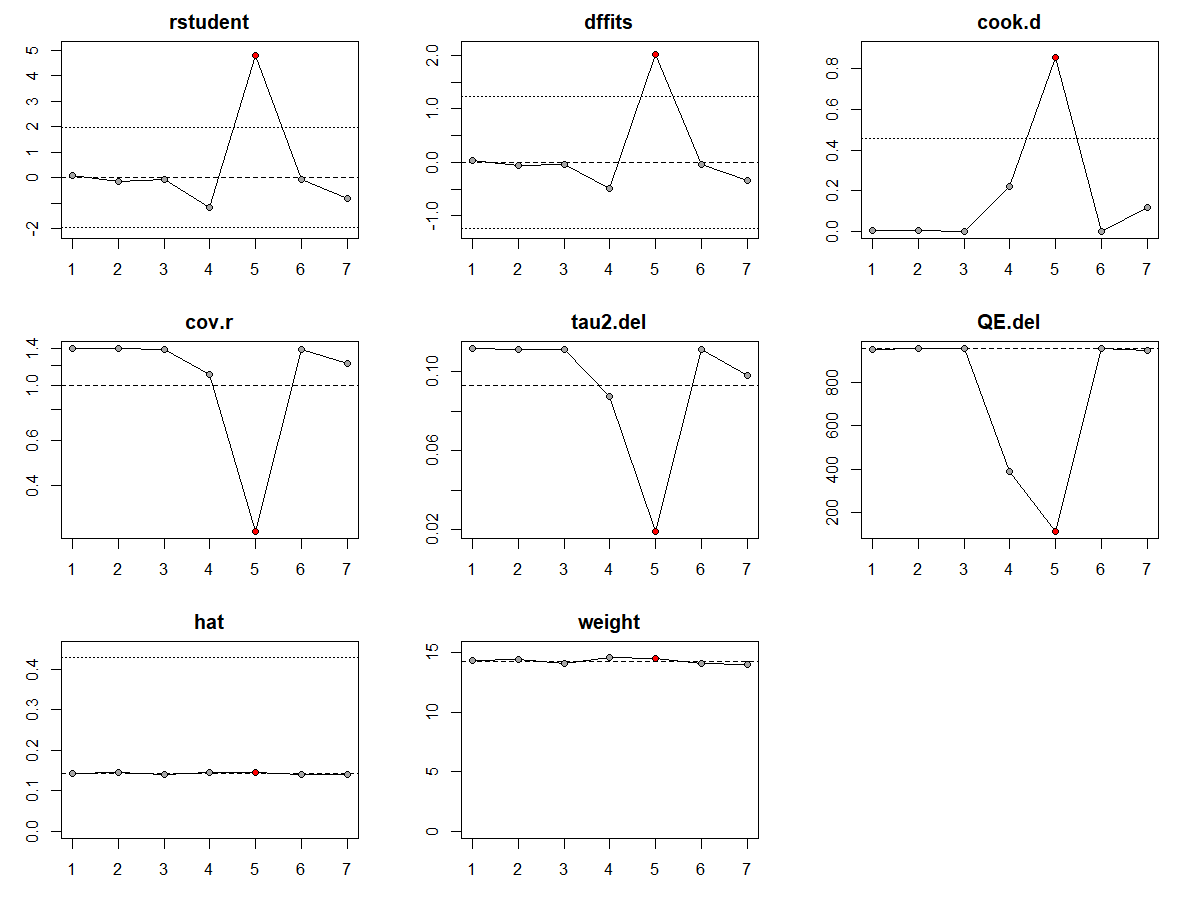


C
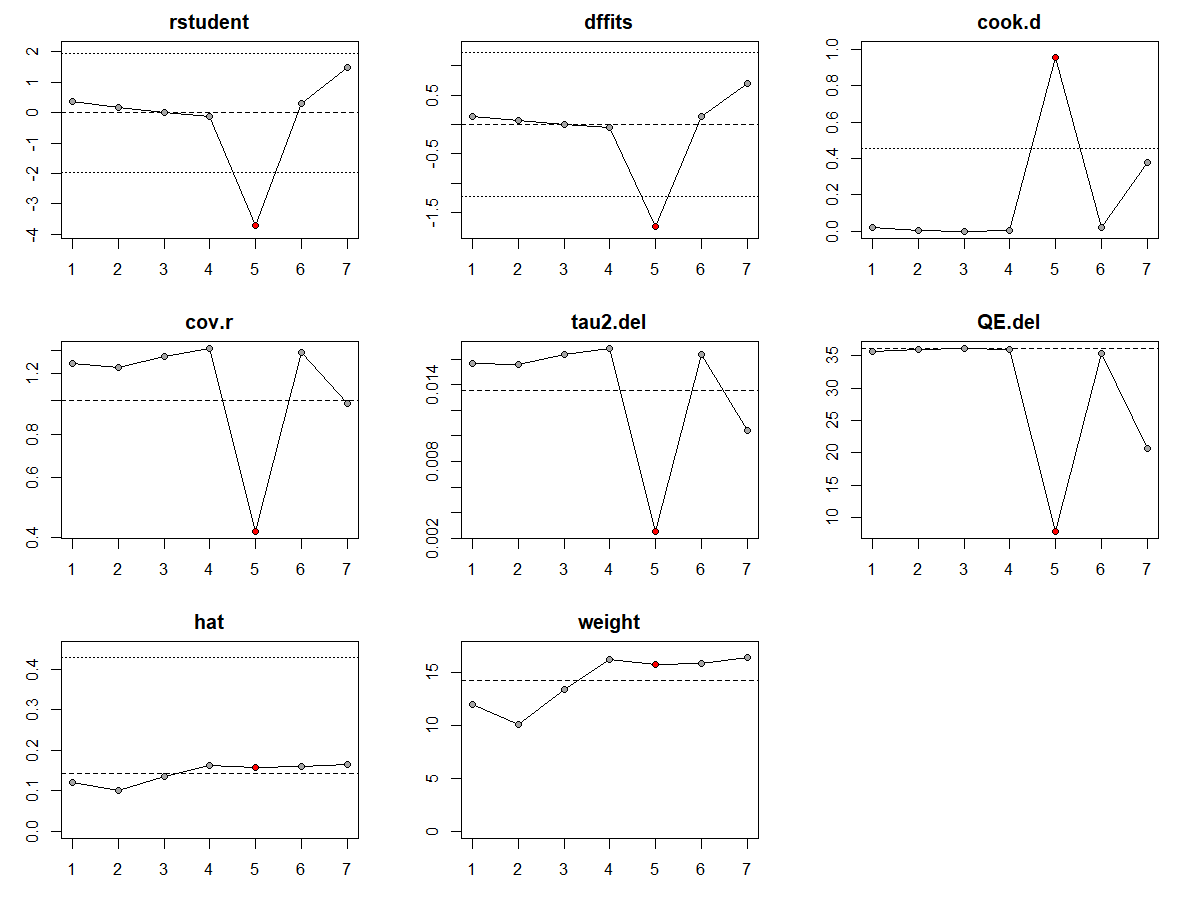
D
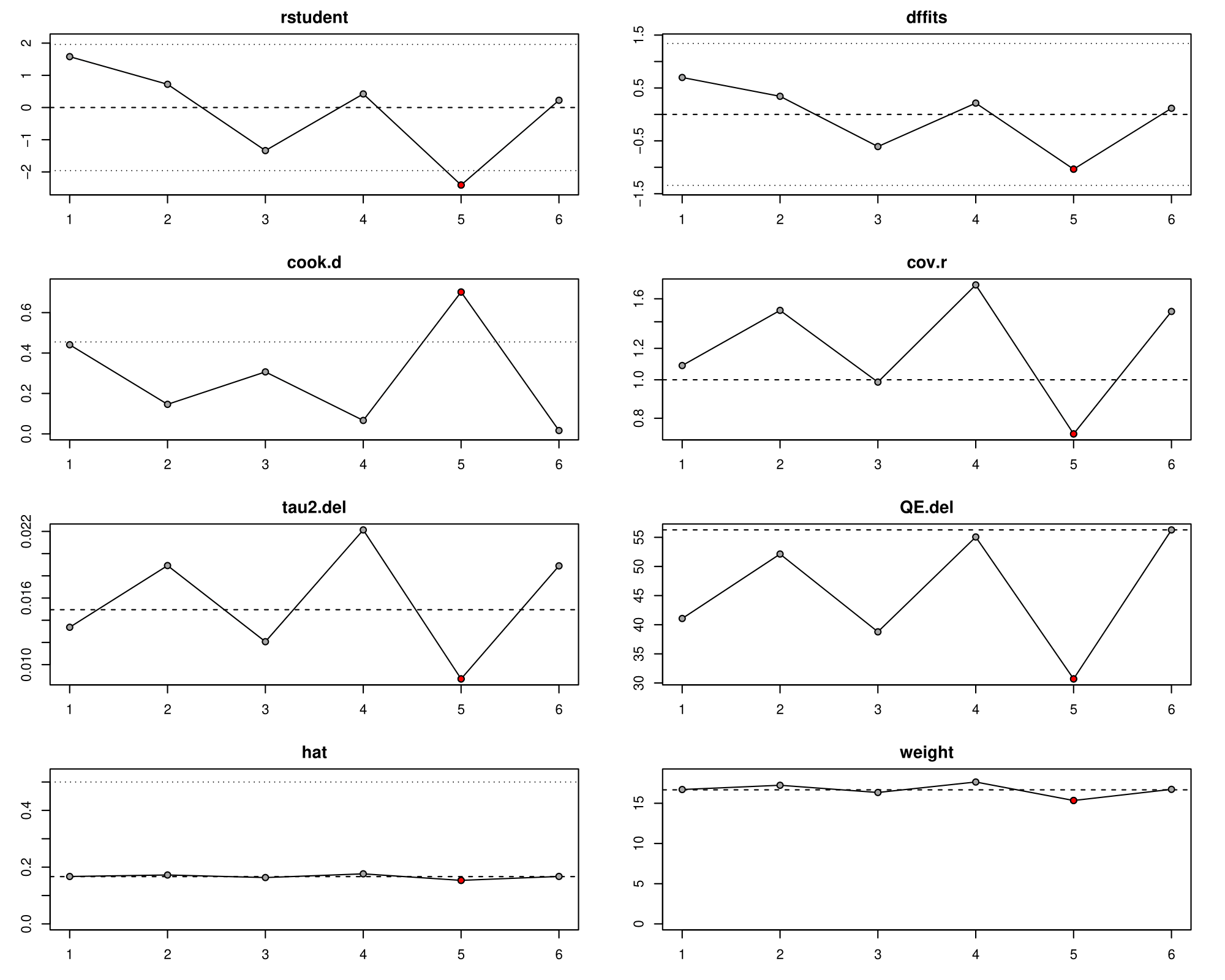


E
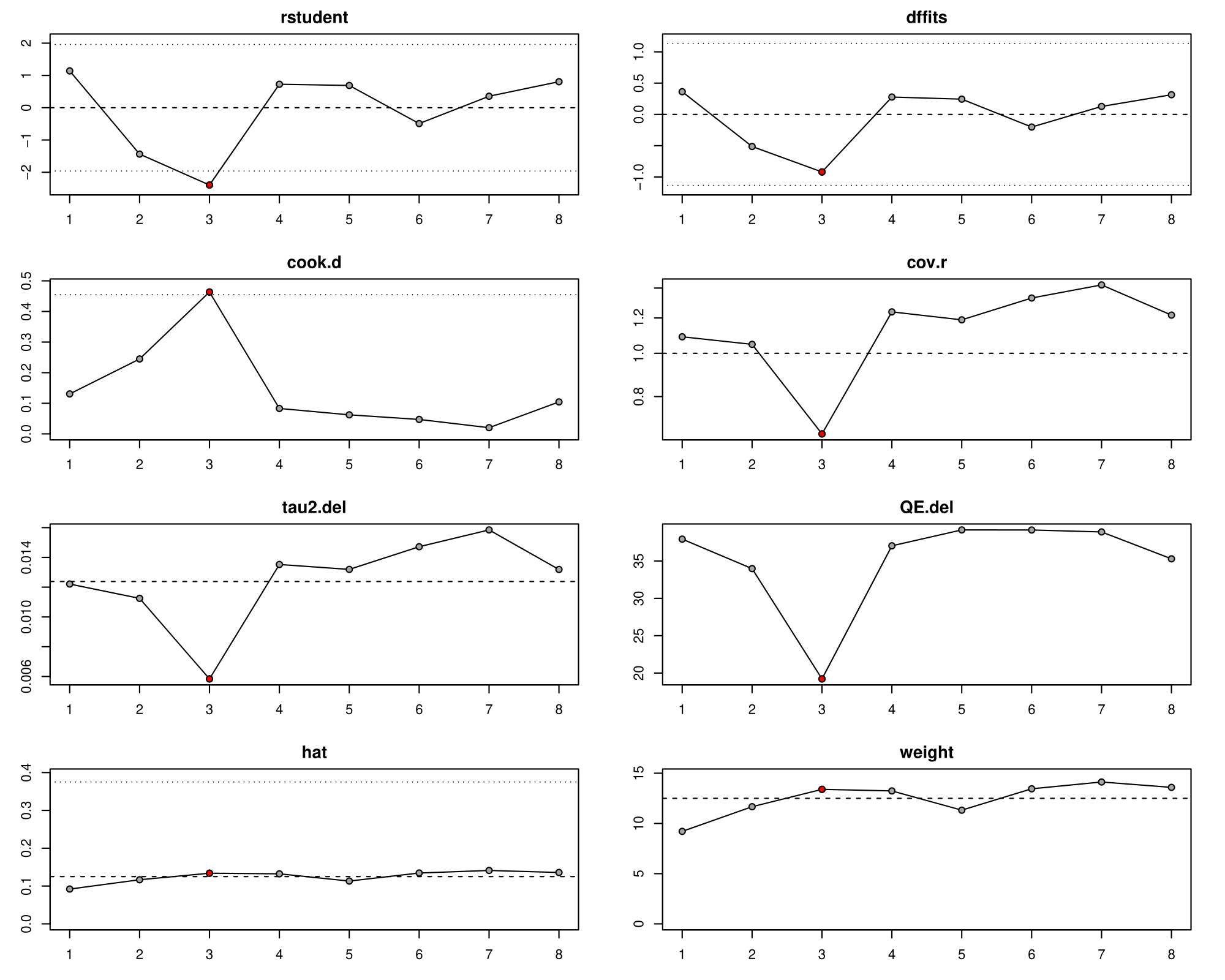


S3. Funnel plots evaluating publication bias for key clinical outcome: (A) NYHA class III–IV postoperative proportion; (B) moderate-to-severe mitral regurgitation (MR ≥ 2); (C) 30-day mortality; (D) long-term mortality; (E) pacemaker implantation. Symmetry of plots indicates low likelihood of publication bias across outcomes.


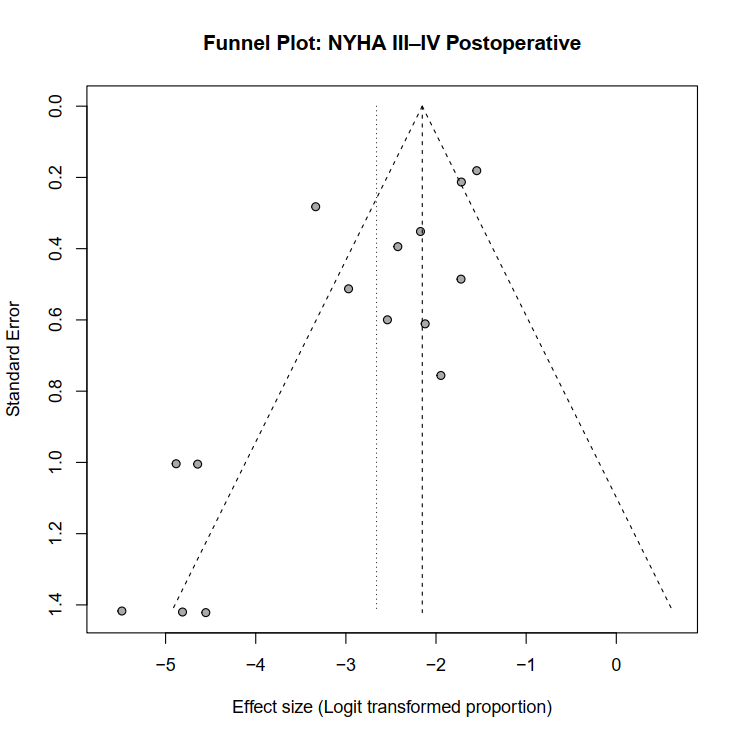

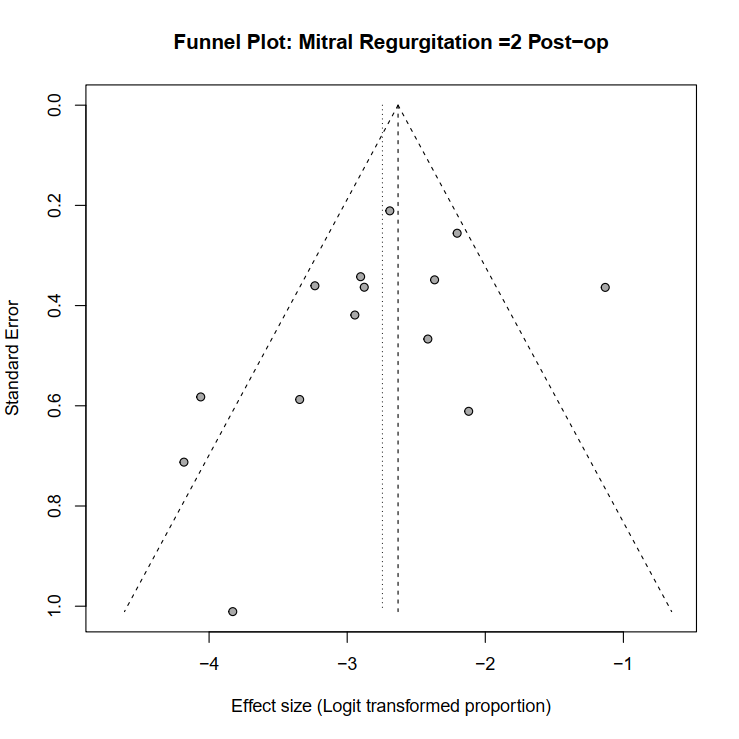


1. (B)


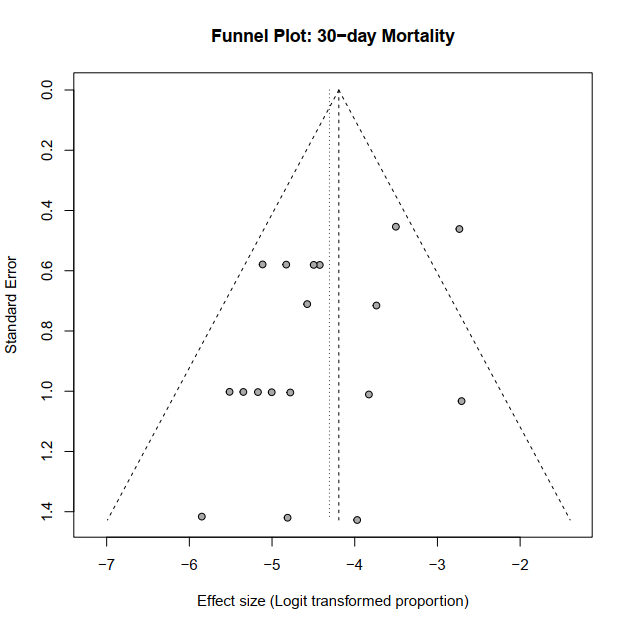

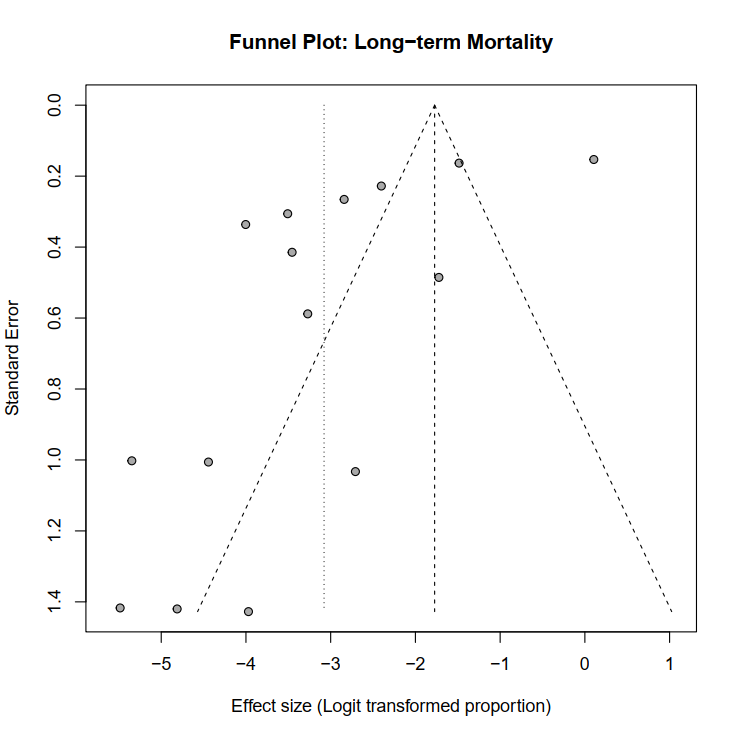


(C) (D)


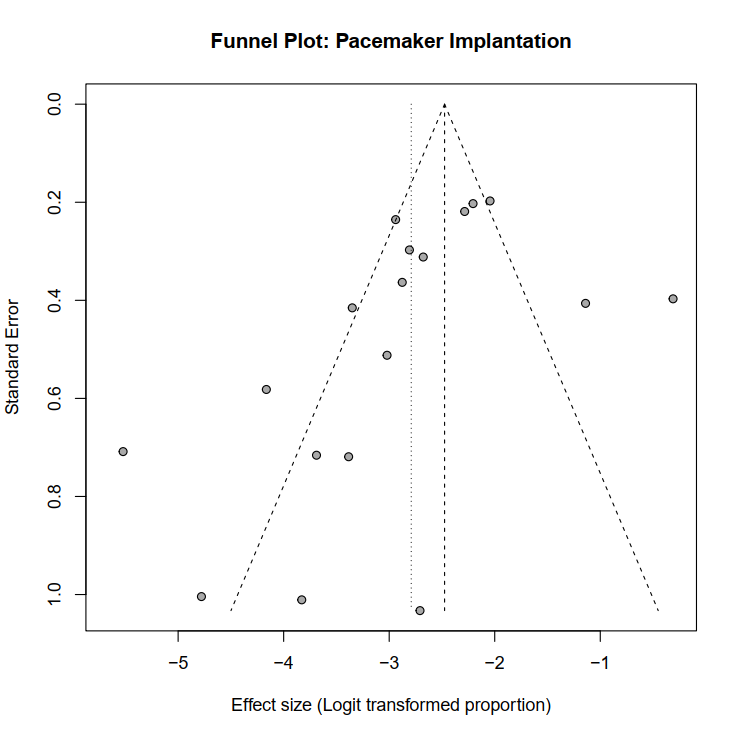


(E)

S4. Meta-regression analysis of clinical predictors and cardiac outcomes: (A) Age vs. NYHA Class Improvement; (B) Follow-up Duration vs. Mitral Regurgitation; (C) Follow-up Duration vs. 30-day Mortality; (D) Residual Mitral Regurgitation vs. Long-term Mortality; (E) Follow-up Duration vs. Long-term Mortality; (F) Follow-up Duration vs. Pacemaker Implantation.

A
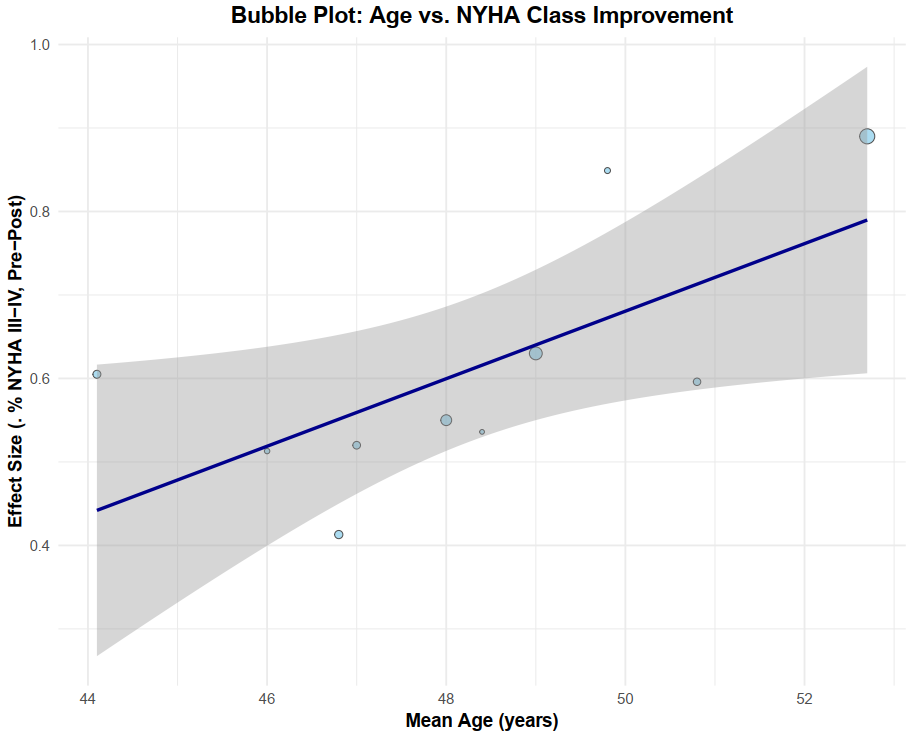
B
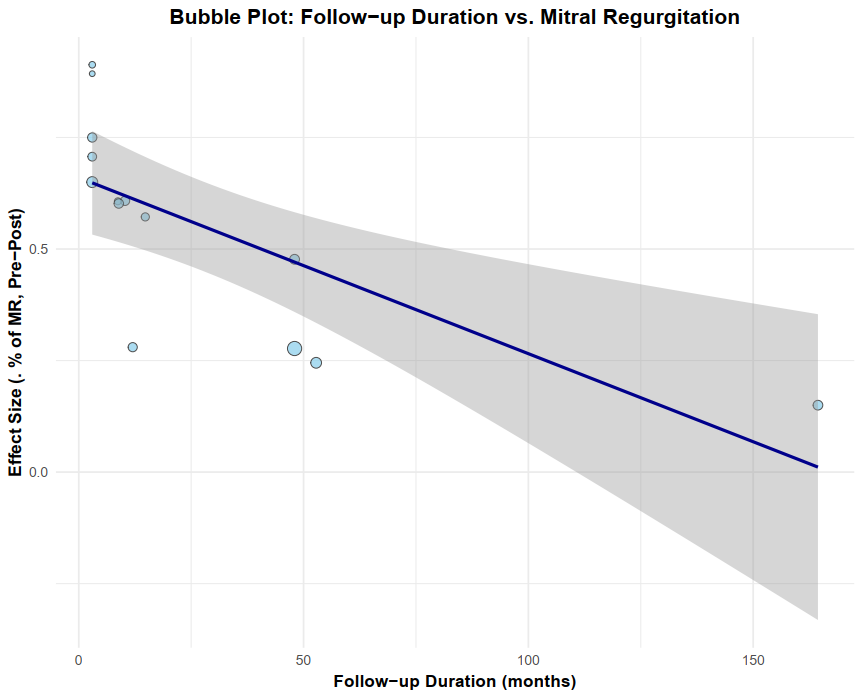


C
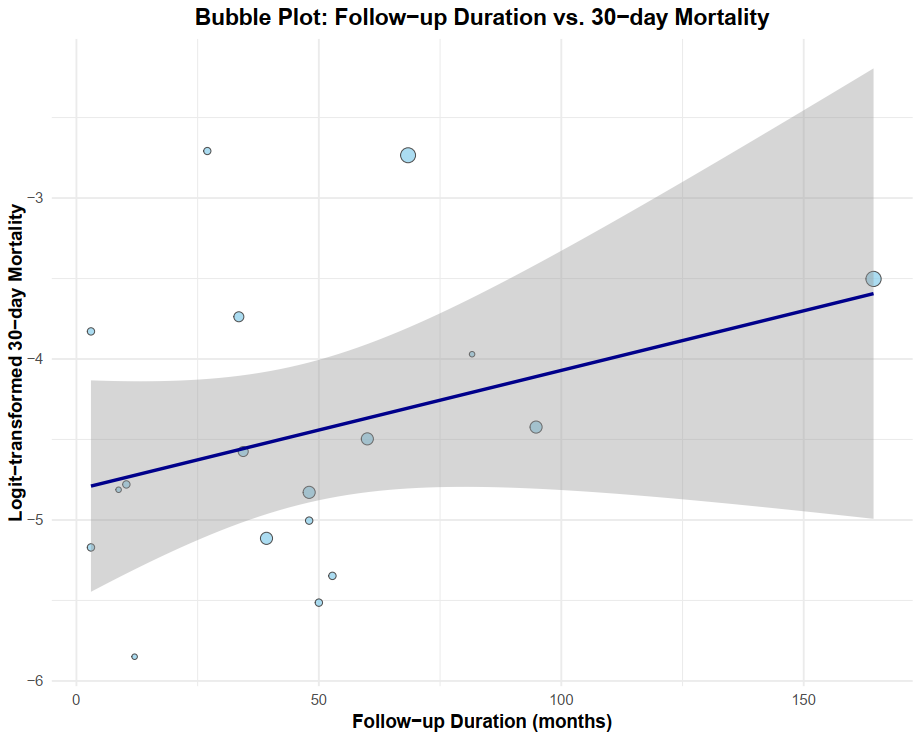
D
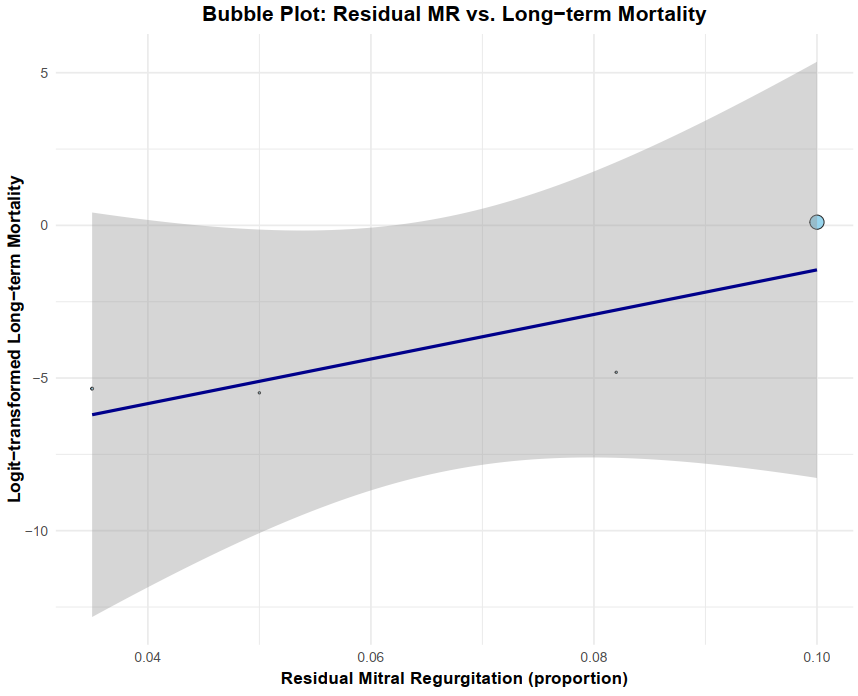


E
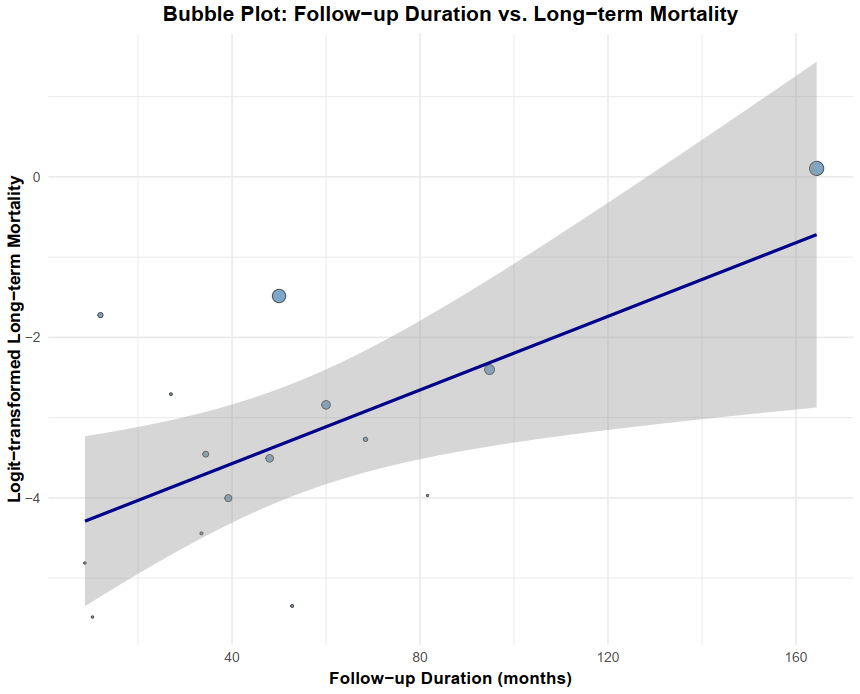
F
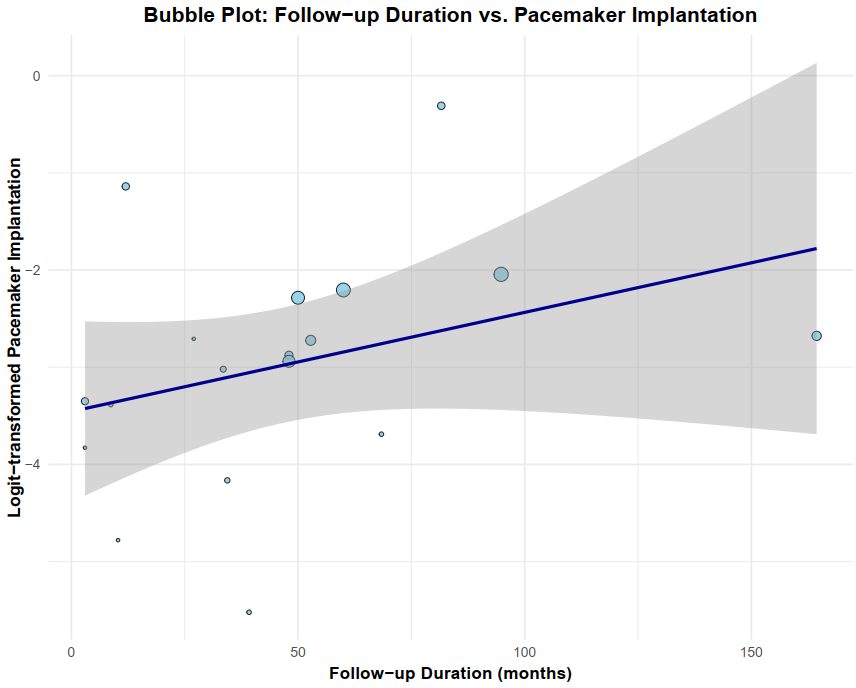

Supplement: Supplementary file 1 [file Table1.docx]
